# Oviposition behaviour of four ant parasitoids (Hymenoptera, Braconidae, Euphorinae, Neoneurini and Ichneumonidae, Hybrizontinae), with the description of three new European species

**DOI:** 10.3897/zookeys.125.1754

**Published:** 2011-08-26

**Authors:** José-María Gómez Durán, Cornelis van Achterberg

**Affiliations:** 1 C/ Corregidor Juan de Bobadilla 2, 8°B, 28030 Madrid, Spain; 2 Department of Terrestrial Zoology, NCB Naturalis, Postbus 9517, 2300 RA Leiden, The Netherlands

**Keywords:** Braconidae, Euphorinae, Neoneurini, *Neoneurus*, *Elasmosoma*, *Kollamosoma*, Ichneumonidae: Hybrizontinae, *Hybrizon*, biology, behaviour, Formicidae, *Formica*, *Cataglyphis*, *Lasius*, new species, key, Europe, Spain, Slovakia, Norway

## Abstract

The oviposition behaviour of four ant parasitoids was observed and filmed for the first time. The movies are available from YouTube (search for *Elasmosoma*, *Hybrizon*, *Kollasmosoma* and *Neoneurus*). Two of the observed species (*Neoneurus vesculus* **sp. n.** and *Kollasmosoma sentum* **sp. n.**) are new to science. A third species (*Neoneurus recticalcar* **sp. n.**) is described from Slovakia and Norway. Keys to the Palaearctic species of the genera *Neoneurus* and *Kollasmosoma* are added.

## Introduction

In Europe the members of the small tribe Neoneurini Bengtsson, 1918 (Hymenoptera: Braconidae: Euphorinae) belong to three genera: *Elasmosoma* Ruthe, 1858, *Kollasmosoma* van Achterberg & Argaman, 1993, and *Neoneurus* Haliday, 1838 (van Achterberg and Argaman 1993). In the past Neoneurini were considered to form a separate subfamily, but recent DNA analyses indicate that it is part of Euphorinae Foerster, 1862 (Belshaw and Quicke 2002; Belshaw et al. 2001).

**Figure 1.**
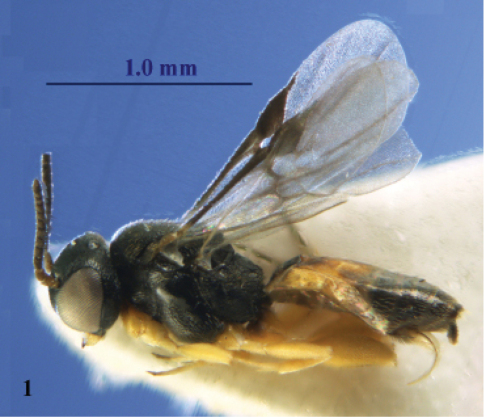
*Elasmosoma luxemburgense* Wasmann, female, Spain, Almazán. Habitus lateral.

As far as the scanty biological information allows a conclusion, the Neoneurini are considered to be most likely koinobiont endoparasitoids of adult ants (Shaw and Huddleston 1991; Shaw 1992). According to Huddleston (1976) the strongly curved ovipositor, which is almost hook-shaped and forward-pointing when exserted (Figs 1, 23, 53), gives support to the supposition that the eggs are laid, possibly through the anus, into the metasoma of adult ant workers. With few exceptions Neoneurini have been found in association with formicine ants (Shenefelt 1969; Marsh 1979; Yu et al. 2007). These ants exude formic acid which is a powerful attractant for predatory ant species, and it seems likely that this exudate could serve also as a kairomonal stimulant to host-seeking hymenopterous parasitoids (Huddleston 1976; van Achterberg and Argaman 1993).

*Elasmosoma* species are predominantly associated with the genus *Formica* Linnaeus, 1758 (*Formica rufa* Linnaeus, 1758, *Formica pratensis* (Retzius, 1783), *Formica sanguinea* Latreille, 1798, *Formica fusca* Linnaeus, 1758, and *Formica rufibarbis* Fabricius, 1793), infrequently also with *Lasius niger* (Linnaeus, 1758), and species of *Camponotus* Mayr, 1861, and *Polyergus* Latreille, 1804, (Wasmann 1897; Schmiedeknecht 1914; Tobias 1971, 1986; Marsh 1979; Huddleston 1976). The observations by Panis (2007) on *Elasmosoma berolinense* in southern France conflict with our observations; he reported oviposition through the intersegmental membrane at the base of the metasoma. Either this is an adaptation to parasitize workers of *Camponotus vagus* (Scopoli, 1763) or the identification of the parasitoid is perhaps incorrect.

The scanty biological information indicates that *Kollasmosoma* species are associated with species of the genus *Cataglyphis* Foerster, 1850. One *Kollasmosoma* species (*Kollasmosoma platamonense* (Huddleston, 1976)) was observed to approach the formicine desert ant *Cataglyphis bicolor* (Fabricius, 1793) from behind and remained in contact with the tip of the metasoma of the ant for less than one second (R.D. Harkness inHuddleston 1976). Similar oviposition behaviour is reported in this paper for another *Kollasmosoma* species (*Kollasmosoma sentum* sp. n.) with *Cataglyphis ibericus* (Emery, 1906) in Spain.

*Neoneurus* species are only associated with members of the genus *Formica* Linnaeus, 1758: *Formica rufa* Linnaeus, *Formica pratensis* (Retzius) and *Formica subsericea* Say, 1836 (reported as *Formica podzolica* Francoeur, 1973; Yu et al. 2007). Tobias (1971) found *Neoneurus auctus* (Thomson, 1895) in bark-beetle galleries (Scolytidae), but this may concern hibernating specimens.

For the identification of the tribe Neoneurini of the subfamily Euphorinae see (van Achterberg (1990, 1993, 1997), for the identification of the Palaearctic genera see van Achterberg and Argaman (1993); for a key to the European *Elasmosoma* species see van Achterberg and Koponen (2003); for references to the genera and species, see Yu et al. (2007) and updates, and for morphological terminology see van Achterberg (1988).

The small subfamily Hybrizontinae Blanchard, 1845 (= Paxylommatinae Foerster, 1862; Wharton and van Achterberg 2000) is considered to belong to the family Ichneumonidae, but was often associated with Braconidae (van Achterberg 1976) or considered to be a separate family (Tobias 1988). The group is treated as a subfamily of the family Ichneumonidae Latreille, 1802, by Rasnitsyn (1980) and Yu and Horstmann (1997), and indeed the structure of the connection of the second and third metasomal tergites and the venation of the hind wing seem to indicate a closer relationship with the family Ichneumonidae (Sharkey and Wahl 1987; Wahl and Sharkey 1988). From analysis of the 28S ribosomal RNA from *Hybrizon* it may be concluded that the Hybrizontinae are at a basal position of the Ichneumonidae-lineage (Belshaw et al. 1998; Quicke et al. 2000; Belshaw and Quicke 2002).

Until now the biology of Hybrizontinae was poorly known and based on circumstantial evidence (van Achterberg 1999). Development was known to take place in ant-nests, from which they have been reared several times and where the naked pupae have been found among ant cocoons (Donisthorpe and Wilkinson 1930). Hybrizontinae are associated with ants of the genera *Formica* Linnaeus, *Lasius* Fabricius, 1804, *Myrmica* Latreille, 1804, and *Tapinoma* Foerster, 1850 (Yu et al. 2007). Donisthorpe (1915) and Donisthorpe and Wilkinson (1930) gave a detailed list of several species of probable hosts based on their own data and that of others (Arnold 1881; Ratzeburg 1848; Giraud 1857; Rudow 1883; Marshall 1891; Wasmann 1894, 1899; Cobelli 1906; de Gaulle 1908; Haupt 1913). According to that list *Hybrizon buccatus* (de Brébisson, 1825) was seen hovering over the nest entrance or over workers of *Formica rufa*, *Formica rufibarbis*, *Formica sanguinea*, *Lasius alienus* (Foerster, 1850), *Lasius brunneus* (Latreille, 1798), *Lasius citrinus* Emery, 1922, *Lasius flavus* (Fabricius, 1782), *Lasius niger*, *Myrmica lobicornis* Nylander, 1846, *Myrmica ruginodis* Nylander, 1846, *Myrmica scabrinodis* Nylander, 1846, and *Tapinoma erraticum* (Latreille, 1798). *Ghilaromma fuliginosi* was seen hovering over *Lasius fuliginosus* (Latreille, 1798). *Eurypterna cremieri* (Romand, 1838) was seen hovering over *Formica rufa*, *Lasius brunneus*, *Lasius fuliginosus* and *Camponotus herculeanus*. More recently, Watanabe (1984) saw *Ghilaromma fuliginosi* (Wilkinson, 1930) hovering over *Lasius fuliginosus*, and Marsh (1989) refers to three specimens of *Hybrizon rileyi* (Ashmead, 1899) which were attracted to a disturbed nest of *Lasius alienus*. In The Netherlands females of *Hybrizon buccatus* were observed diving at *Formica rufa* worker ants during spring ant wars in the dunes near The Hague (van Achterberg 1999). In total, four genera of ants (*Formica*, *Lasius*, *Myrmica* and *Tapinoma*) belonging to three subfamilies (Formicinae, Myrmicinae and Dolichoderinae) have been considered as probable hosts of *Hybrizon buccatus*. This large range of host-parasitoid relationships needs, beyond the mere existence of hovering behaviour, direct confirmation of ovipositions. In the case of *Eurypterna cremieri*, Cobelli (1906) observed 20 ovipositions on the larvae transported by workers of *Lasius fuliginosus*, and Komatsu and Konishi (2010) photographed oviposition into larvae of *Lasius nipponensis* Forel, 1912. From the circumstantial evidence it was concluded that they probably are endoparasitoids of ants (Čapek 1970; van Achterberg 1976). Here we report that the egg of *Hybrizon buccatus* is laid in ant larva when they are transported outside of their nests. Komatsu and Konishi (2010) report oviposition by *Eurypterna cremieri* into the somatic cavity of the larva in less than a second. Konishi (2010) stated that the larvae are parasitized during their transport from the summer nest in a tree trunk to the winter ground nest in October; the adults of *Eurypterna cremieri* emerge from the summer nest in the tree trunk from late September to end of October. It remains a mystery how this large parasitoid manages to get enough food considering that the host is half the size of the parasitoid. In addition, Komatsu and Konishi (2010) described and illustrated oviposition by a new Japanese genus and species into larvae of *Myrmica kotokui* Forel, 1911. For the recognition of the subfamily Hybrizontinae and for the identification of European genera and species, see Tobias (1988) and van Achterberg (1999).

## Material and methods

Females of *Hybrizon buccatus* (de Brébisson) and *Elasmosoma luxemburgense* Wasmann were observed in Almazán (Soria, Spain) in July and August, 2010. *Kollasmosoma sentum* sp. n. and *Neoneurus vesculus* sp. n. were observed in Madrid (at the enclosed area of the Institute for Agriculture and Food Research and Technology (INIA), Carretera de La Coruña Km 7.5, Spain) in August and September, 2010. The oviposition behaviour for each species (comprising the grasping of the ant by the wasp and the insertion of the ovipositor, until departure by flight) was recorded in slow motion video, at a rate of 300 frames per second, with a Casio Exilim Pro EX-F1 digital camera and a Raynox DCR-250 Super Macro lens. The four short movies showing the oviposition behaviour of the four observed species are downloadable from YouTube (Appendices I–IV). RMNH stands for Netherlands Centre for Biodiversity Naturalis, Leiden, Netherlands; RMS for National Museums of Scotland, Edinburgh, RMSEL for Rocky Mountain Systematic Entomology Laboratory, Laramie, Wyoming, USA, and ZMUO for Zoological Museum, University of Oslo, Blindern, Oslo, Norway.

## Braconidae Nees, 1811

Elasmosoma **Ruthe, 1858**

*Elasmosoma* Ruthe, 1858: 7. Type species by monotypy: *Elasmosoma berolinense* Ruthe, 1858 (examined).

### Elasmosoma luxemburgense

Wasmann, 1909

http://species-id.net/wiki/Elasmosoma_luxemburgense

Figs 1 [Fig F2] [Fig F3] [Fig F4] [Fig F5] [Fig F6] –7

#### Oviposition behaviour

Oviposition of *Elasmosoma* spp. into the ants’ metasoma has long been observed (Forel 1874; Olivier 1893; Pierre 1893; Wasmann 1897; Donisthorpe 1927; Kariya 1932), and adults of *Elasmosoma* have been reared from *Formica* nests on various occasions (Wasmann 1897; Watanabe 1935; Poinar 2004). Due to the very quick act of oviposition, few details are known about the accompanying behaviour of grasping the ant or about the location of ovipositor insertion. Wasmann (1897) supposed that *Elasmosoma* females lay the eggs between the abdominal segments; other authors, considering the strongly curved morphology of the ovipositor, have suggested that the eggs are probably laid through the anus (Huddleston 1976; van Achterberg and Argaman 1993). Here we report new observations on the oviposition behaviour of *Elasmosoma luxemburgense* on *Formica rufibarbis* Fabricius, 1793, comprising alighting and grasping the worker ant and ovipositor insertion.

The observations were made in Almazán (Soria, Spain) in August, 2010 on a warm and calm day between 12.26 PM and 13.38 PM. A group of 30 to 40 of *Formica rufibarbis* workers were present surrounding a nest entrance on the ground. They were excited and aggressive, carrying materials, entering and leaving the nest. Some cadavers of another species of ant and isolated fights indicated that a more extensive battle recently occurred. Forel (1874) noted that these struggle situations attract *Elasmosoma berolinense*, and possibly the formic acid exuded in the course of these fights serve as a kairomon to the parasitoid wasps (Huddleston, 1976). During the 72 minutes of observation, groups of 2–3 females of *Elasmosoma luxemburgense* could be seen hovering over and attacking the ants at a height of 1–3 cm from the ground. A total of 50 attempts at oviposition was recorded (Movie *Elasmosoma*, Appendix I). The ants were aware of these attacks, turning around and chasing the wasps with open mandibles. On one occasion, a worker caught a wasp while flying (Movie *Elasmosoma*, last sequence).

**Figure 2. F2:**
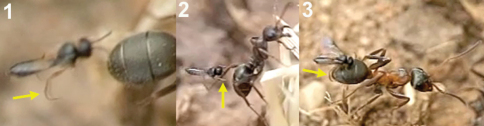
**1** female of *Elasmosoma luxemburgense* approaches the ant’s metasoma with the hind legs extended in curved shape (arrow) **2** the fore legs are darted forward (arrow) **3** when alighting the hind legs brace the apex of the ant’s metasoma (arrow).

The wasp attacks always come from behind, paralleling their longitudinal axis to those of the ants. When they are less than 1 cm from an ant they dart forward and the fore legs contact the dorsal surface of the metasoma first. Meanwhile the hind legs, arranged in curved shape, are situated to brace the apex of the metasoma (Fig. 2).

**Figure 3. F3:**
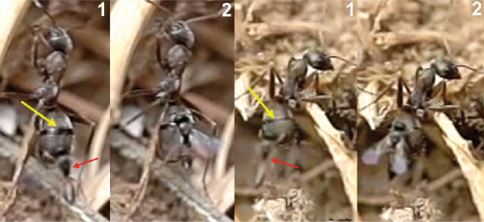
Two sequences of a female of *Elasmosoma luxemburgense* (red arrow) hitting on the posterior margin of the first gastral segment (yellow arrow) of *Formica rufibarbis*. After hitting, the wasp begins to fold its wings.

Contact with the fore legs is usually followed by hitting of the parasitoid’s head on the host’s metasoma. At this moment the middle and hind legs grasp the metasoma and the wasp folds its wings. The site chosen by the wasp for the initial hit of the fore legs, or the head, is usually the posterior margin of the first gastral segment (T1; Fig. 3), i.e., of a total of 48 hits observed, 44 were on the posterior margin of the first gastral segment (91.7%), three on the posterior margin of the second (6.3%), and one on the posterior margin of the third (2%).

**Figure 4. F4:**
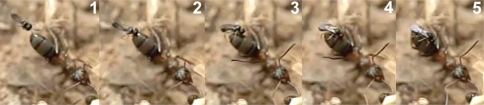
**1** female of *Elasmosoma luxemburgense* approaches the ant metasoma **2** hits on the posterior margin of the third gastral segment **3** begins to climb **4** arrives at the posterior margin of the second gastral segment **5** reaches the posterior margin of the first gasrtal segment.

When the hit occurs at the posterior margin of the second or third gastral segments, the wasp climbs onto the metasoma, changing its position to reach the posterior margin of the first gastral segment (Fig. 4).

**Figure 5. F5:**
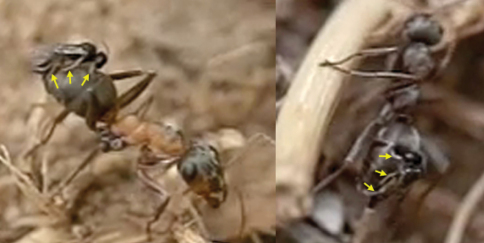
Arrangement of the legs of *Elasmosoma luxemburgense* grasping the ant’s metasoma for oviposition.

This locational preference for alighting may be visually stimulated by the differentiated border of the posterior margin of the first gastral segment, enhanced by the characteristic dark stripe behind it. The frame analysis in the film clip suggests that the wasp’s head hits the posterior margin of T1 with the mandibles opened, and that a slight deformation of the suture between T1 and T2 is produced. Presumably, the modified structure of the T1-T2 suture is used by the wasp to secure its grasp. The tarsal modifications of *Elasmosoma* (vestigial tarsal claws and enlarged pulvillus; Shaw 1985, 2007) may be adaptations to effect this grasping behaviour. In the final arrangement, prior to oviposition, the fore tarsi usually grasp the posterior margin of the first gastral segment, and the hind tibiae and tarsi brace the apex of the metasoma on the fourth gastral segment, with the middle legs positioned near or somewhat posterior to the hind margin of the second gastral segment (Fig. 5).

This arrangement of the legs facilitates the appropriate position of the wasp’s metasoma in order to insert the ovipositor into the posterior area of the last metasomal segment, between the pygidium and the hypopygium, probably through the anus. Poinar (2004) dissected the metasoma of the ant *Formica obscuriventris clivia* Creighton, 1940, a host of *Elasmosoma michaeli* Shaw, 2007, and found for the first time the wasp egg “just under the body wall of the ant’s metasoma.”

**Figure 6. F6:**
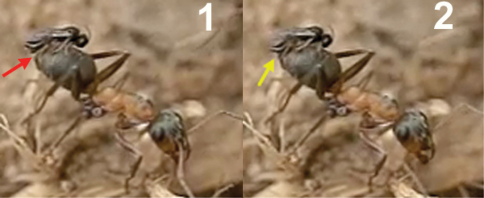
Insertion of the ovipositor by *Elasmosoma luxemburgense*. **1** the red arrow shows the wasp’s metasoma separated from the ant’s metasoma **2** the yellow arrow shows the metasoma of the parasitoid and of the ant joined during insertion of the wasp’s ovipositor. The fore legs have now advanced their position towards the posterior margin of the first gastral segment.

The precise moment of ovipositor insertion could be detected by the conspicuous downward-movement of the apex of the wasp’s metasoma (Fig. 6 and first sequence of Movie *Elasmosoma*). Although one single movement of the apex of the metasoma normally occurred during oviposition, in some cases 2 or 3 consecutive movements were observed. On one occasion the same wasp alighted and oviposited two consecutive times in the same ant.

**Figure 7.**
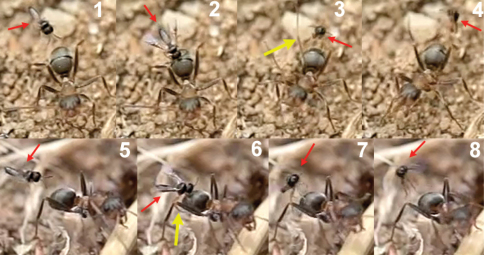
Two sequences of failed attacks by *Elasmosoma luxemburgense*. **1** the wasp (red arrow) approaches the ant **2** the wasp hits the metasoma **3** the right hind leg of the ant (yellow arrow) strikes the wasp and **4** throws it off **5** the wasp approaches the ant **6** when alighting, the right hind leg of the wasp (yellow arrow) remains over the hind leg of the ant, impeding the grasp of the ant’s metasoma **7** and **8** the wasp flies away.

Oviposition attempts sometimes failed due to strong movements of the ant’s metasoma, to strikes by the ant’s legs, or because of defective alighting by the wasp (Fig. 7). Of a total of 50 oviposition attempts, 40 were successful (80%) and 10 failed (20%). The whole oviposition behaviour of *Elasmosoma luxemburgense* (comprising grasping of the ant by the wasp and the insertion of the ovipositor, until taking off) lasted a mean of 0.727 seconds (95% confidence interval: 0.578–0.877; N = 38; SE = 0.074), with a median of 0.602 seconds (interquartile range: 0.480–0.900) (Fig. 79).

### Kollasmosoma

van Achterberg & Argaman, 1993

http://species-id.net/wiki/Kollasmosoma

Fig. 8

Kollasmosoma van Achterberg & Argaman, 1993: 66. Type species by original designation: *Elasmosoma platamonense* Huddleston, 1976 (examined).

#### Key to species of the genus *Kollasmosoma* van Achterberg & Argaman

**Table:** 

1	Scapus longer than pedicellus, somewhat longer than wide (Fig. 29); third antennal segment somewhat longer than fourth segment (Fig. 29); fore tarsus shorter than middle tarsus; face, frons and vertex transversely striate; hypopygium of female protruding apically; face of female black; C. Asia (Kazakhstan)	*Kollasmosoma marikovskii* (Tobias, 1986)
–	Scapus (excluding radix) shorter than pedicellus, wider than long (Figs 11, 24, 34); third antennal segment distinctly shorter than fourth segment (Figs 11, 24, 34); fore tarsus 1.3–1.9 times as long as middle tarsus (Fig. 39); face, frons and vertex granulate; hypopygium of female (sub)truncate apically (Figs 14, 37); face of female pale yellowish or white, of male black (males only known of *Kollasmosoma sentum*)	2
2	Dorsal face of propodeum distinctly longer than metanotum, similar to posterior face (Fig. 26); inner spur of hind tibia of female enlarged and apically truncate (and outer spur acute; Fig. 25); basitarsus of middle leg about 1.5 times as long as second tarsal segment (Fig. 27); [temple about 0.7 times as wide as eye in lateral view (Fig. 61, l.c.)]; Mongolia	*Kollasmosoma cubiceps* (Huddleston, 1976)
–	Dorsal face of propodeum about as long as metanotum or shorter (Figs 10, 37); inner spur of hind tibia of female normal and apically acute (Figs 13, 33); basitarsus of middle leg 2–3 times as long as second tarsal segment (Fig. 28); Mediterranean; Iberian Peninsula	3
3	Outer spur of hind tibia of female enlarged and apically obtuse (Fig. 33); fifth metasomal sternite of female without apical spine (Fig. 37); face moderately convex (Fig. 37); height of eye of female about 4.8 times width of temple in lateral view (Fig. 37); dorsal face of propodeum about as long as metanotum (Fig. 37); pedicellus of female less protruding and scapus much wider than long (Fig. 34); fore tarsus of female about 1.3 times as long as middle tarsus; East Mediterranean	*Kollasmosoma platamonense* (Huddleston, 1976)
–	Outer spur of hind tibia of female normal and apically acute (Fig. 13); fifth metasomal sternite of female with an apical spine (Fig. 14); face strongly convex (Fig. 9); height of eye of female about 3.6 times width of temple in lateral view (Fig. 9), of male about 2.8 times; dorsal face of propodeum shorter than metanotum (Fig. 10); pedicellus (= second antennal segment) of female more protruding and scapus slightly wider than long (Fig. 11), but much shorter in male; fore tarsus of female about 1.9 times as long as middle tarsus (of male about 1.2 times); West Mediterranean (Iberian Peninsula)	*Kollasmosoma sentum* van Achterberg & Gómez, sp. n.

### Kollasmosoma sentum

van Achterberg & Gómez sp. n.

urn:lsid:zoobank.org:act:F4B69A39-303E-40D3-BE04-C2A076307DCD

http://species-id.net/wiki/Kollasmosoma_sentum

Figs 8 [Fig F9] [Fig F10] [Fig F11] [Fig F12] [Fig F13] [Fig F14] [Fig F15] [Fig F16] [Fig F17] –23

#### Type material.

Holotype, ♀ (RMNH),“Spain, Madrid, Carretera de La Coruña km 7.5, 20.viii.2010, following adult workers of *Cataglyphis ibericus*, J.M. Gómez Durán, RMNH”. Paratypes: 7 ♀ (RMNH (5), RMSEL (1)), topotypic, collected 3.ix. and 13.ix.2010; 1 ♂ (RMS), “(Spain), Granada, Orgiva, 3OS VF68, 500 m, 11241”, “Leg. Jose Luis Ruiz de la Cuesta, 6.v.2009, 11241”. The only known male paratype of *Kollasmosoma platamonense* from Spain probably also belongs here.

#### Oviposition behaviour.

Few observations have been made on the biology of the small Palaeartic parasitoid genus *Kollasmosoma* van Achterberg & Argaman, 1993. *Kollasmosoma platamonense* is known to approach the ant *Cataglyphis bicolor* from behind, briefly contacting its metasoma (R.D. Harkness in Huddleston 1976); van Achterberg and Argaman (1993) reported this species hovering over the nest of *Messor semirufus*, but no oviposition was observed. *Kollasmosoma marikovskii* has been reared from *Formica pratensis* (van Achterberg and Argaman, 1993) and, finally, no information is available on the biology of *Kollasmosoma cubiceps* (Huddleston). Here we report some observations on oviposition by *Kollasmosoma sentum* sp. n. in the ant *Cataglyphis ibericus* (Emery, 1906). The observations were made in Madrid (at the enclosed area of the Institute for Agriculture and Food Research and Technology (INIA), Carretera de La Coruña Km 7.5, Spain) during August and September, 2010. The parasitized colony of *Cataglyphis ibericus* had a polycalic nest with three entrances on the ground, forming a triangle of about 60 cm on each side. This area was visited daily by females of *Kollasmosoma sentum* during the three weeks of observation. The wasps appeared in groups of 1–3 individuals at any time between 12 PM and 15.30 PM, in the hours of highest temperature (around 35° Celsius). The visits lasted between 30 and 90 minutes. The wasps hovered over the nest entrances or looked for worker ants in the surrounding area when going out to forage or when returning to the nest carrying prey (thus, walking slowly). The wasps’ attacks usually occurred during the brief and characteristic stops of *Cataglyphis* ants when marching. The wasp was extremely fast, flying at a height of about 1 cm over the ground. In order to observe and record the wasp’s oviposition behaviour, the very speedy workers of *Cataglyphis ibericus* were kept quiet by means of baits such as *Messor barbarus* cadavers -a usual prey of this species- and honey (Fig. 15).

When the wasp approaches, the ant is often aware of its presence, aggressively turning around with opened mandibles, or extending the hind or middle legs to hit the wasp even if it comes from behind (Fig. 16). This defensive behaviour is very common and sometimes prevents the wasp from alighting and ovipositing.

*Kollasmosoma sentum* attacks the ant from behind, and oviposition takes place into both the dorsal and ventral surface of the ant’s metasoma, more rarely into its apex (Movie *Kollasmosoma*, Appendix II). (On one exceptional occasion, a wasp was observed attacking the ant’s head). In all the cases observed (n= 22) the movements of the wasp’s metasoma during oviposition, and hence the insertion of the ovipositor, followed the direction of the postero-anterior axis of the ant’s metasoma, which suggests that the ovipositor may be inserted through an intersegmental membrane. Basically, two alighting strategies have been observed for achieving the postero-anterior insertion of the ovipositor; strategies that depend on the flight direction of the wasp’s attack and on the inclination of the ant’s metasoma, this last varying from an horizontal position to a vertical one, perpendicular to the ground surface and distinctive for the genus *Cataglyphis*.

1) Horizontal alighting: the wasp follows an ant with its metasoma in, or near, a horizontal position, approaches it from behind, in the direction of the longitudinal axis of the ant, and extends the fore legs until grasping the dorsal metasomal surface with its tarsi. With this grasp the wasp jumps over the ant’s metasoma, lays down the middle and hind legs, and folds its wings before starting to oviposit (Fig. 17).

2) Vertical alighting: the wasp follows an ant having its metasoma arranged in vertical position, or forming an angle bigger than 45 degrees with the ground surface. It approaches the ant from behind, sometimes following a direction deviating from the longitudinal axis of the ant, and extends its fore legs until grasping the ventral metasomal surface with the tarsi. Now, with this grasp, the wasp accomplishes two kinds of rotational movements, which vary according to both the flight direction of the wasp and the inclination of the ant’s metasoma. An example of this surprising pirouette, that fully involves the two rotations, occurs when the wasp, in horizontal flight, approaches an ant’s metasoma placed in a vertical position (Fig. 18 and the first two sequences of Movie *Kollasmosoma*). After grasping the ant’s metasoma with the tarsi, and being perpendicularly aligned with respect to it, the wasp starts a 180° rotation around its longitudinal axis. At the same time, the wasp rotates vertically, approaching the metasoma. As a result of both rotational movements, the wasp alights downwards, allowing it to insert the ovipositor following the direction of the postero-anterior axis of the ant’s metasoma.

It is interesting that during the rotation movements of the wasp, its fore tarsi (Fig. 19) keep permanent contact with the ant’s metasoma. To achieve rotation around its longitudinal axis (without lifting the legs off), the tarsi are placed slightly separated, one over the other, on the ventral surface of the ant’s metasoma (Figs 20 and 21). If the right tarsus is placed over the left one, the wasp rotates counter clockwise; if the left tarsus is placed over the right one, the rotation is clockwise. This longitudinal disposition of the wasp’s tarsi on the ant’s metasoma is, therefore, a behavioural adaptation to enable the necessary rotation of the body before oviposition.

The rapid insertion of the ovipositor follows a uniform behavioural pattern. When alighting, the wasp grasps the ant’s metasoma with its three pairs of legs and folds its wings. Immediately, the wasp moves gradually backwards toward a perpendicular position with respect to the metasoma surface, the apex of its metasoma remaining over the ant’s metasoma. A good example is offered during horizontal alighting (Fig. 22): the body of the wasp goes back tending to the vertical position. Before reaching the vertical, the apex of the wasp’s metasoma moves down, presumably inserting the ovipositor into the ant’s metasoma. At the vertical position, the apex of the wasp’s metasoma presses down on the ant’s metasoma, completely attaching to it. The wasp continues leaning backwards some way beyond the vertical and, finally, takes flight backwards.

Regarding the oviposition behaviour of *Kollasmosoma sentum* sp. n., the probable function of the ventral spine, peculiar to this species, located on the fifth sternite (anterior to the hypopygium; Fig. 23) needs mention. Since the rapid insertion of the ovipositor occurs when the wasp is in or near a perpendicular position with respect to the surface of the ant’s metasoma (most likely with the fore legs detached from it), the ventral spine could serve to fix the wasp’s position and act as a supporting point for the oviposition movements of the wasp’s metasoma.

The whole oviposition behaviour of *Kollasmosoma sentum* sp. n. (comprising the grasping of the ant by the wasp and the insertion of the ovipositor, until flight; Fig. 79) lasted a mean of 0.052 seconds (95% confidence interval: 0.047–0.057; N = 19; SE = 0.002), with a median of 0.050 seconds (interquartile range: 0.047–0.057).

#### Diagnosis.

Outer spur of hind tibia of female normal and apically acute (Fig. 13); fifth metasomal sternite of female with an apical spine (Figs 14, 23); face strongly convex (Fig. 9); height of eye about 3.6 times width of temple in lateral view (Fig. 9); dorsal face of propodeum shorter than metanotum (Fig. 10); pedicellus of female distinctly protruding (Fig. 11); fore tarsus of female about 1.9 times as long as middle tarsus.

#### Description.

Holotype, ♀, length of body 2.0 mm, of fore wing 1.4 mm.

*Head.* Length of third segment of antenna 0.5 times fourth segment, length of third, fourth and penultimate segments 0.5, 0.8 and 1.0 times their width, respectively, and basal segments with distinct setae; pedicellus distinctly protruding and larger than scapus; face strongly convex and densely setose (Fig. 9), without facial tubercles and bristles; length of eye 2.4 times temple in dorsal view; height of eye about 3.6 times width of temple in lateral view (Fig. 9); vertex superficially granulate and having a satin sheen; temples roundly narrowed behind eyes; OOL:diameter of ocellus:POL = 5:4:20; length of malar space 0.05 times height of eye, eye nearly touching base of mandible.

*Mesosoma.* Length of mesosoma 1.1 times its height; mesoscutum evenly granulate; scutellum granulate and distinctly convex; precoxal sulcus absent; mesopleuron superficially granulate, but speculum shiny and largely smooth; mesosternal sulcus narrow and micro-crenulate; metanotum without a median carina and longer than dorsal face of propodeum; propodeum finely rugulose, dorsal face much shorter than posterior face, with satin sheen, without a median carina and no medial areola and its spiracle small and far in front of middle of propodeum.

**Figure 8.**
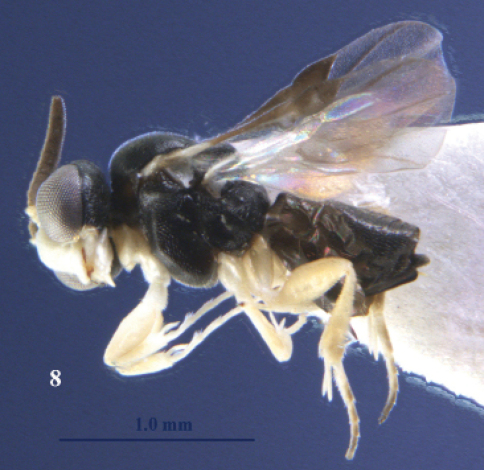
*Kollasmosoma sentum* sp. n., female, holotype. Habitus lateral.

*Wings.* Fore wing: parastigma comparatively large (Fig. 8); vein SR distinctly pigmented; basal half of wing much less densely setose than its distal half. Hind wing: wing membrane sparsely setose basally.

*Legs.* Hind coxa partly superficially micro-granulate, nearly smooth and with satin sheen; fore coxa nearly flat ventrally; all tarsal claws slender and simple; length of femur, tibia and basitarsus of hind leg 2.9, 4.5 and 4.0 times their width, respectively; fore femur moderately curved in dorsal view, compressed and apically without tooth; fore tibia without protuberances and evenly densely setose, its length 6.3 times its maximum width in lateral view; fore tarsus 1.9 times as long as middle tarsus and 1.6 times as long as fore tibia; fore tibial spur slightly curved and 0.7 times as long as fore basitarsus and 0.4 times fore tibia (Fig. 12); spurs of hind tibia acute apically, their length 1.1 and 1.0 times hind basitarsus.

*Metasoma.* Length of first tergite 0.6 times its apical width, its surface with satin sheen, granulate, basally and medially flat, and its spiracles not protruding and near apex of tergite; second and third tergites superficially granulate; second metasomal suture obsolescent; remainder of metasoma largely smooth and depressed; fifth sternite with a large and acute apical spine (Fig. 14); setae of metasoma spread and short; second tergite with sharp lateral crease; length of ovipositor sheath 0.05 times fore wing.

*Colour.* Black; face, clypeus, labrum, malar space, frons antero-laterally and medially, palpi, propleuron, tegula, wings basally, fore and middle legs white; scapus and pedicellus, and hind leg ivory, but hind tarsus dorsally infuscate; pronotal side with brown patch; veins brown; remainder of antenna, humeral plate largely, metasoma laterally, parastigma and pterostigma largely dark brown; wing membrane subhyaline.

*Variation.* Length of body 1.8–2.1 mm, of fore wing 1.1–1.4 mm, all females have 12 antennal segments; pronotal side may be largely brown.

#### Etymology.

From “sentus” (Latin for “thorny, spiny”), because of the unique thorn-like spine of the fifth sternite of the female.

**Figures 9–14. F9:**
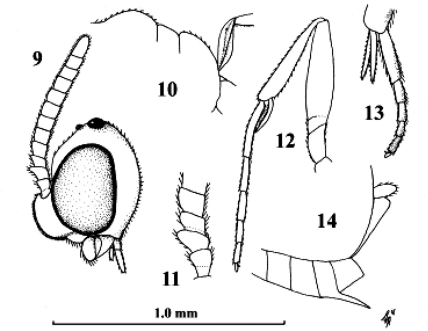
*Kollasmosoma sentum* sp. n., female, holotype. **9** head lateral **10** profile of posterior half of mesosoma **11** base of antenna lateral **12** fore leg lateral inner side **13** hind tarsus and tibial spurs lateral **14** apex of metasoma lateral. Scale-line = 1.0×, but of 11 1.5×.

**Figure 15. F10:**
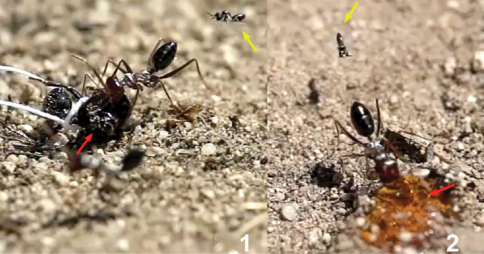
Baits were used to keep the ants quiet **1** *Messor barbarus* cadavers (red arrow) tied with a thread and fixed to the ground **2** honey (red arrow). Females of *Kollasmosoma sentum* sp. n. are indicated with a yellow arrow.

**Figure 16. F11:**
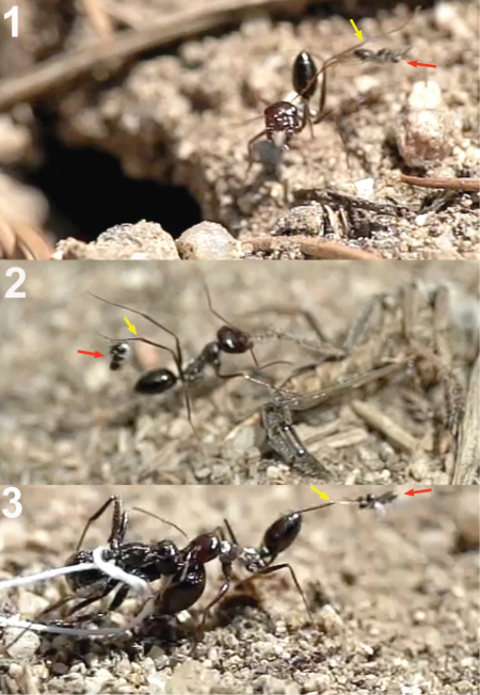
Workers of *Cataglyphis ibericus* hitting females of *Kollasmosoma sentum* sp. n. (red arrow) with its legs (yellow arrow). **1** at the nest entrance **2** and **3** at the baits.

**Figure 17. F12:**
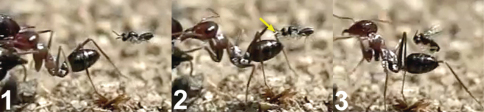
Horizontal alighting. **1** female of *Kollasmosoma sentum* sp. n. approaches an ant with the metasoma in horizontal position **2** extends the fore legs (yellow arrow) and grasps the metasoma with the tarsi **3** jumps over the metasoma placing the rest of its legs on it, and folds its wings.

**Figure 18. F13:**
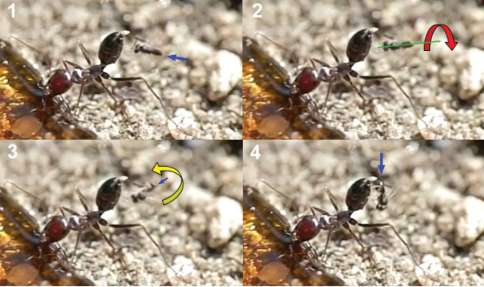
Vertical alighting. **1** female of *Kollasmosoma sentum* sp. n. (blue arrow) grasps the ant’s metasoma with its fore tarsi **2** starts a 180° rotation around its longitudinal axis **3** at the same time initiates a second rotation, moving vertically towards the ant’s metasoma **4** alights downwards on the ant’s metasoma.

**Figure 19. F14:**
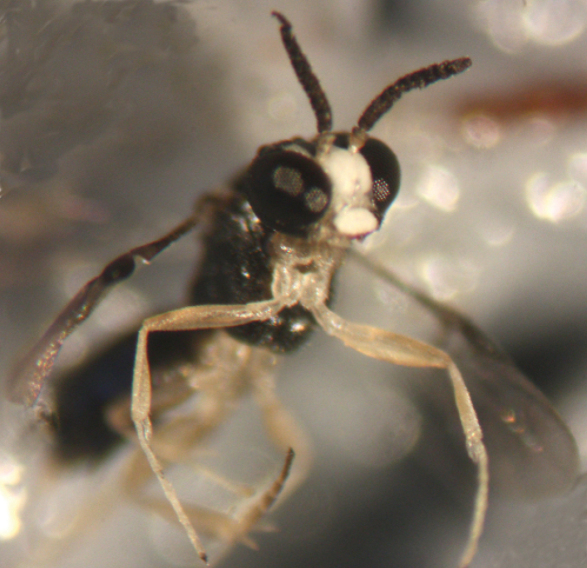
Stereomicroscopic image showing the fore legs of a female of *Kollasmosoma sentum* sp. n.

**Figure 20. F15:**
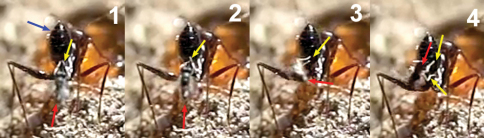
Arrangement of the fore legs a female *Kollasmosoma sentum*sp. n. for the rotation movement. **1** The wasp (the red arrow points the apex of its metasoma) approaches the ant’s metasoma (blue arrow) and extends its fore legs (yellow arrow) **2** the right tarsus is placed over the left one **3** the wasp starts its counter clockwise rotation (yellow arrow points to separation between the fore legs) **4** the wasp alights downwards; at that moment the hind and middle legs (yellow arrow) grasp the ant’s metasoma, and the fore legs move forwards.

**Figure 21. F16:**
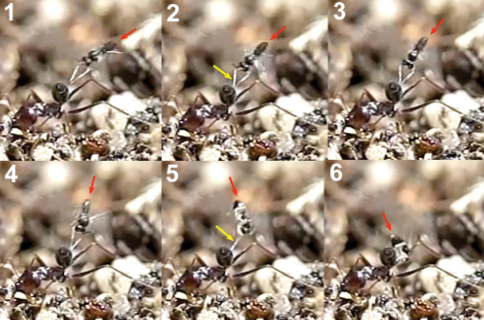
Arrangement of the fore legs of the female of *Kollasmosoma sentum* sp. n. for the rotation movement. **1** the wasp (the red arrow points the apex of the metasoma) approaches the ant’s metasoma and extends its fore legs **2** the right tarsus (yellow arrow) is placed over the left one **3** and **4** the wasp starts its counter clockwise rotation around its longitudinal axis **5** the wasp is in profile and the right fore leg hides the left one **6** the wasp alights downwards on the ant’s metasoma.

**Figure 22. F17:**
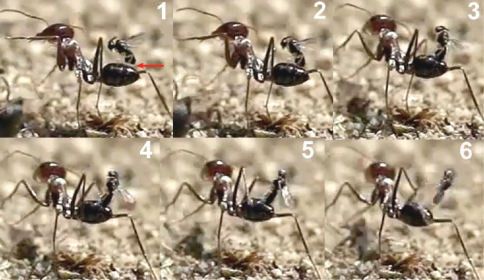
Oviposition of *Kollasmosoma sentum* sp. n. **1** After alighting and folding its wings, the wasp begins to lean backwards (the red arrow points to the space between the apex of the wasp’s metasoma and the ant’s metasoma) **2** before reaching the vertical, the apex of the wasp metasoma moves down, presumably inserting the ovipositor **3** at the vertical position, the apex of the wasp’s metasoma is completely attached to the ant’s metasoma **4** and **5** the wasp continues leaning backwards **6** the wasp flies off backwards.

**Figure 23.**
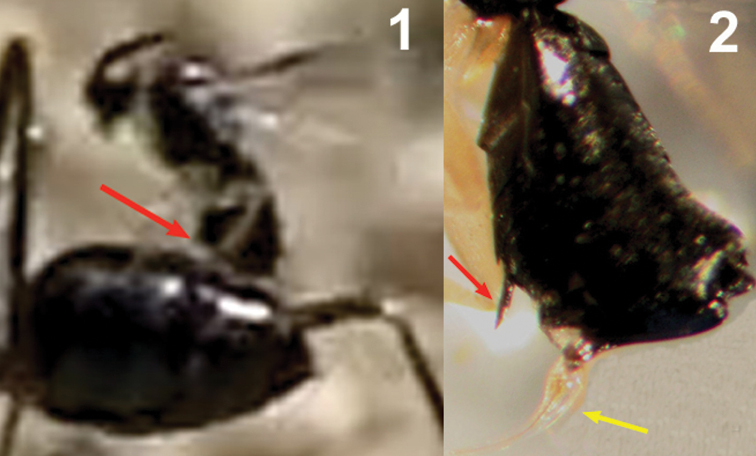
**1** Detail of the moment of oviposition of *Kollasmosoma sentum* sp. n. showing the location of the ventral spine (red arrow) **2** stereomicroscopic image showing the ventral spine (red arrow) and the exserted ovipositor (yellow arrow).

**Figures 24–27.**
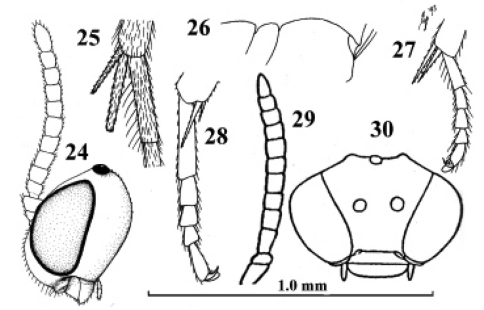
*Kollasmosoma cubiceps* (Huddleston), female, paratype. **28.** *Kollasmosoma platamonense* (Huddleston), female, holotype. **29–30.** *Kollasmosoma marikovskii* (Tobias), female, holotype. **24** head lateral **25** hind tibial spurs lateral **26** profile of posterior half of mesosoma **27 28** middle tarsus and tibial spurs lateral **29** antenna lateral **30** head anterior. 24 scale-line = 1.0×, 25–28 1.4×, 29 30 after Tobias (1986).

**Figures 31–43.**
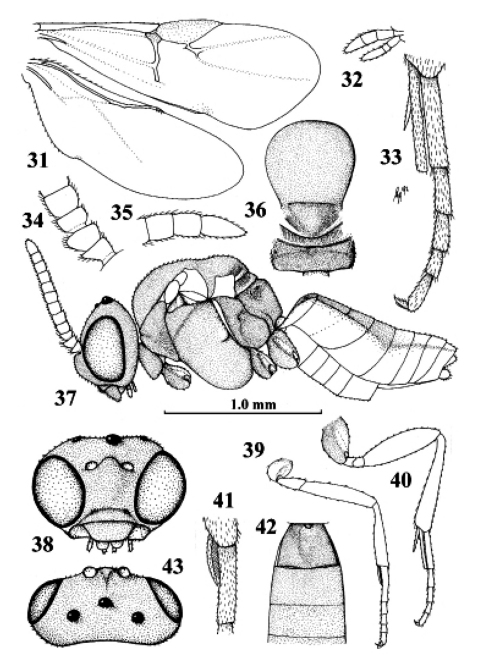
*Kollasmosoma platamonense* (Huddleston), female, Israel, Eilot. **31** wings **32** palpi **33** hind tibial spurs **34** base of antenna **35** apex of antenna **36** mesosoma dorsal **37** habitus lateral **38** head anterior **39** fore leg **40** hind leg **41** fore tarsal spur **42** firstthird metasomal tergites dorsal **43** head dorsal. 31 36–40 42 43: scale-line = 1.0×, 32–35 41: 2.2×.

### Neoneurus

Haliday, 1838

http://species-id.net/wiki/Neoneurus

Fig. 44

Neoneurus Haliday, 1838: 213 (no species). Type species (= first species included by Marshall (1897): *Neoneurus halidaii* Marshall, 1897 (examined; = *Neoneurus auctus* (Thomson, 1895); examined).

#### Key to Palaearctic species of the genus *Neoneurus* Haliday

**Table:** 

1	Females: third and following antennal segments with short and adpressed setae; fore tibia widened subbasally (Figs 46, 49); metasoma apically with a strongly downcurved ovipositor and a short and elliptical ovipositor sheath (Fig. 53)	2
–	Males: third and following antennal segments with medium-sized and erect setae; fore tibia narrow subbasally (Figs 47, 50); ovipositor and ovipositor sheath absent	7Note. Males are unknown of the C. Asian *Neoneurus curvicalcar* Belokobylskij, 1986, and the European *Neoneurus vesculus*sp. n. and *Neoneurus recticalcar* sp. n.
2	Fore femur straight in dorsal view (Fig. 45) and comparatively narrow in lateral view (Fig. 46); face without facial tubercles and bristles (Fig. 48); fore tibia without protuberances (Fig. 46); fore basitarsus 0.7–0.9 times as long as fore tibia (Fig. 46); [fore tibial spur 0.3–0.5 times hind basitarsus and its apical half narrow (Fig. 46); vertex finely transversely striate or rugulose; tegulae pale yellowish; anterior subalar prominence pale dark brown; pedicellus brown, darker than yellowish scapus; only European species with entirely black or dark brown metasoma]; W & E Palaearctic	*Neoneurus auctus* (Thomson, 1895)
–	Fore femur curved in dorsal view (Figs 51, 56, 59, 69) and comparatively wide in lateral view (Figs 50, 58, 70); face with pair of facial tubercles and a central bristle (Figs 52, 57, 60, 62, 67); fore tibia with protuberances (Figs 49, 55, 58, 63, 68); fore basitarsus about 0.5 times as long as fore tibia and apical half gradually narrowed (Figs 49, 55, 58)	3
3	Anterior subbasal tooth of fore tibia minute (Figs 49, 55); posterior longitudinal carina of fore tibia short (Figs 49, 55)	4
–	Anterior subbasal tooth of fore tibia wide triangular (Figs 58, 68); posterior longitudinal carina of fore tibia long, surpassing middle of tibia (Fig. 58)	5
4	Fore tibial spur nearly straight and 0.7–0.8 times as long as fore basitarsus (Fig. 55); facial tubercles minute, with slender bristle and distance between bristles 1.2–1.3 times width of scapus (Fig. 57); apical tooth of fore femur medium-sized (Fig. 56); fore tibia 4.5–5.0 times as long as wide (Fig. 57); mesopleuron sometimes partly pale yellowish medially; W Palaearctic	*Neoneurus recticalcar* van Achterberg, sp. n.
–	Fore tibial spur strongly curved and 0.8–0.9 times as long as fore basitarsus (Fig. 49); facial tubercles medium-sized, with robust bristle and distance between bristles about equal to width of scapus (Fig. 52); apical tooth of fore femur minute (Fig. 51); fore tibia about 6 times as long as wide (Fig. 49); mesopleuron dark brown medially; W & E Palaearctic	*Neoneurus clypeatus* (Foerster, 1862)
5	Posterior longitudinal carina of fore tibia with a submedial thorn-like protuberance; facial tubercles thick and nearly as long as pedicellus; [fore tibial spur distinctly curved in both sexes]; E Palaearctic (Mongolia)	*Neoneurus armatus* Tobias, 1977
–	Posterior longitudinal carina of fore tibia without a submedial thorn-like protuberance (Figs 58, 68); facial tubercles shorter than pedicellus (Figs 60, 67)	6
6	Pair of facial bristles minute, 0.2 times as long as pedicellus and distance between bristles about 1.2 times width of scapus (Fig. 60); posterior subbasal tooth of fore tibia small, narrow (Fig. 58); SW Palaearctic	*Neoneurus vesculus* van Achterberg & Gómez, sp. n.
–	Pair of facial bristles medium-sized, about as long as pedicellus and distance between bristles about equal to width of scapus (Fig. 67); posterior subbasal tooth of fore tibia medium-sized, wide triangular or falcate (Fig. 68); E Palaearctic (Kazakhstan)	*Neoneurus curvicalcar* Belokobylskij, 1986
7	Hind femur partly dark brown or black; fore tibial spur strongly curved; [vertex finely granulate; clypeus black]; Mongolia	*Neoneurus armatus* Tobias, 1977
–	Hind femur yellowish-brown or brown, sometimes infuscate basally; fore tibial spur moderately curved or nearly straight (Figs 47, 50)	8
8	Length of fore tarsus 1.8–2.0 times fore tibia and tibia widened apically (Fig. 47); fore tibial spur straight or nearly so and with medium-sized setae (Fig. 47); clypeus with satin sheen and transversely striate; epistomal suture obsolescent laterally; vertex dorsally transversely striate or rugulose; W & E Palaearctic	*Neoneurus auctus* (Thomson, 1895)
–	Length of fore tarsus 1.2–1.5 times fore tibia and tibia slender apically (Fig. 50); fore tibial spur moderately curved and with short setae (Fig. 50); clypeus shiny and smooth or nearly so; epistomal suture distinct laterally; vertex dorsally finely granulate or coriaceous; W & E Palaearctic	*Neoneurus clypeatus* (Foerster, 1862)

### Neoneurus auctus

(Thomson, 1895)

http://species-id.net/wiki/Neoneurus_auctus

Figs 44 –48

#### Synonyms.

*Neoneurus halidaii* Marshall, 1897 (examined); *Euphorus bistigmaticus* Morley, 1909 (synonymised by Shaw (1992)).

#### Material.

(all RMNH unless otherwise indicated) Austria (Aschbach, 1400 m; Bach, Lechtal, 1200 m), Bulgaria (Rogen; h. Teneran; Batak; Smoljanski esera; h. Erqupria; Jemkovo; m. Nektenica; Gababovo (all Rhodopi)), England (RMS: Midhurst Common, W. Sussex, hovering above and swooping down on *Formica rufa* L., with short abdominal contact observed; Ascot, Berkshire), Finland (Enontekiö, Lappland; Kangaslampi (RMS); Sb, Savonranta, Muhamäki, in window trap on dead aspen (Museum Helsinki), France (Pèzénar), Germany (Ottmaring, Bayern), Netherlands (Otterlo, with *Formica nigricans* Emery, 1909 (= *Formica pratensis* Retzius, 1783); ‘t Harde; Naarden; Oostkapelle, Oranjezon), Norway (Bvardalen, flying over *Formica aquilonia* Yarrow; Sandfjellet, Bergen, id.; Lom, Lia, Oppland), Scotland (RMS: Morrone Birkwood, Aberdeenshire), Sweden (Romelson, Västerbotten (RMS)), Ukraine (Kanev). Mainly collected in May to early August, one specimen from Bulgaria was collected at the end of August. France and Netherlands are new records.

#### Notes.

One male from Bulgaria (RMNH: H. Ruen, Rhodopi Mts, 29.vii.1969, A. Germanov) belongs to a related species; it has the fore tarsus 1.5 times as long as fore tibia, the fore tibial spur with rather long setae and straight and the vertex granulate.

**Figure 44.**
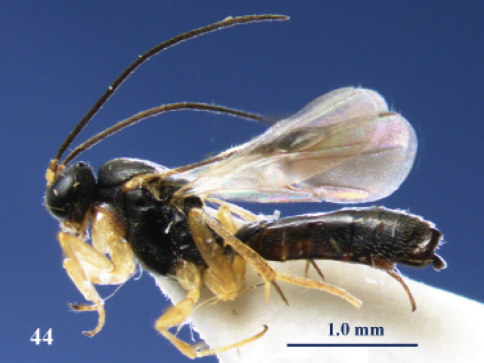
*Neoneurus auctus* (Thomson), female, Netherlands, Oostkapelle. Habitus lateral.

### Neoneurus clypeatus

(Foerster, 1862)

http://species-id.net/wiki/Neoneurus_clypeatus

Figs 49 –53

#### Material.

Netherlands (Meijendel, dunes near The Hague; Rockanje, wet *Salix repens* dunes; Oostkapelle, Oranjezon; ‘t Harde). Mostly collected in August and September, but a few specimens were collected in May and June.

#### Synonyms.

*Elasmosoma viennense* Giraud, 1871, syn. n. (examined). According to Shaw (1992) the holotype of *Neoneurus clypeatus* is a male and has the third and fourth antennal segments shorter and less densely setose and the hind tibial spurs and hind tarsal segments shorter than in the male holotype of *Elasmosoma viennense*; the body, hind coxa and the antenna of the holotype of *Neoneurus clypeatus* are much paler than those of *Elasmosoma viennense*. *Elasmosoma viennense* has the tegulae pale yellowish; dark brown or brown, the clypeus yellowish-white, yellowish-brown or brown and dorsally darkened, the hind coxa yellowish; largely black or dark brown. However, the holotype of *Neoneurus clypeatus* is coloured as a typical female and it may be either just an anomaly or a female. In general, Foerster types are paler by ageing than types from other collections of similar age, including Giraud types and limited weight should be given to colour differences in general.

#### Notes.

 If the scapus and tegulae of a male are pale yellowish or whitish, the hind coxa largely yellowish-brown and the vertex finely granulate, the specimen may represent the unknown male of *Neoneurus vesculus* or *Neoneurus recticalcar*, the latter probably has a slenderer fore femur and tibia than the former.

**Figures 45–48.**
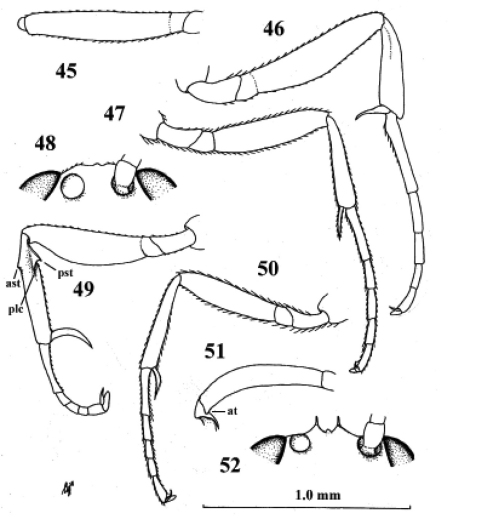
*Neoneurus auctus* (Thomson), female, Netherlands, Oostkapelle, but **47** male, Norway, Lom. **49–52.** *Neoneurus clypeatus* (Foerster), female, Netherlands, Meijendel, but **50** male of same locality**. 45–51** fore femur dorsal **46, 47, 49, 50** fore leg inner side lateral **48, 52** face dorsal. **ast** anterior subbasal tooth **at** apical tooth **plc** posterior longitudinal carina.

**Figure 53.**
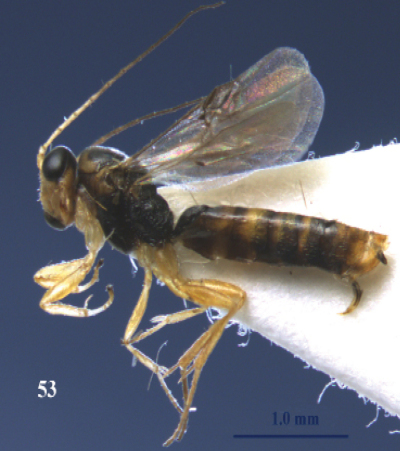
*Neoneurus clypeatus* (Foerster), female, Netherlands, ‘t Harde. Habitus lateral.

### Neoneurus recticalcar

van Achterberg sp. n.

urn:lsid:zoobank.org:act:FCFCE816-B847-4552-AAAF-5842B22A0138

http://species-id.net/wiki/Neoneurus_recticalcar

Figs 54 –57

#### Type material.

Holotype, ♀ (RMNH),“Slovakia, Predna Hora, n[ea]r Murán, 25.vii-1.viii.2009, 850 m, 48°46’N, 20°06’E, Mal. traps, Schacht tr. [trap on southern face of sandy hill], C. v. Achterberg, RMNH’09”. Paratypes: 2 ♀ (RMNH), same label data and EMT; 1 ♀ (ZMUO), “Norway, EIS 46, HES, Elverum, Starmoen NR [N], 11.vi.-29.vii.2004, UTM 32V WGS84, PN 4624 4907, L.O. Hansen/ E. Rindal, Malaise trap N: sandy pine forest”.

#### Oviposition behaviour.

Unknown.

#### Diagnosis.

**Figure 54.**
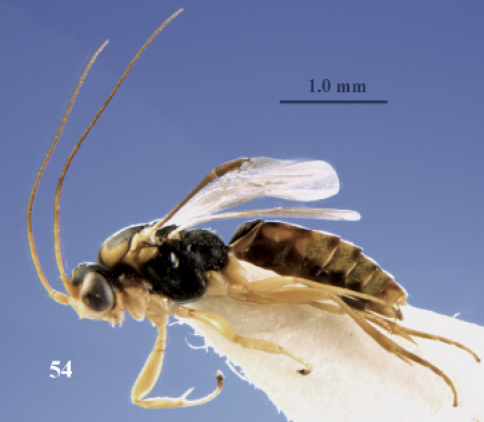
*Neoneurus recticalcar* sp. n., female, holotype. Habitus lateral.

**Figures 55–57.**
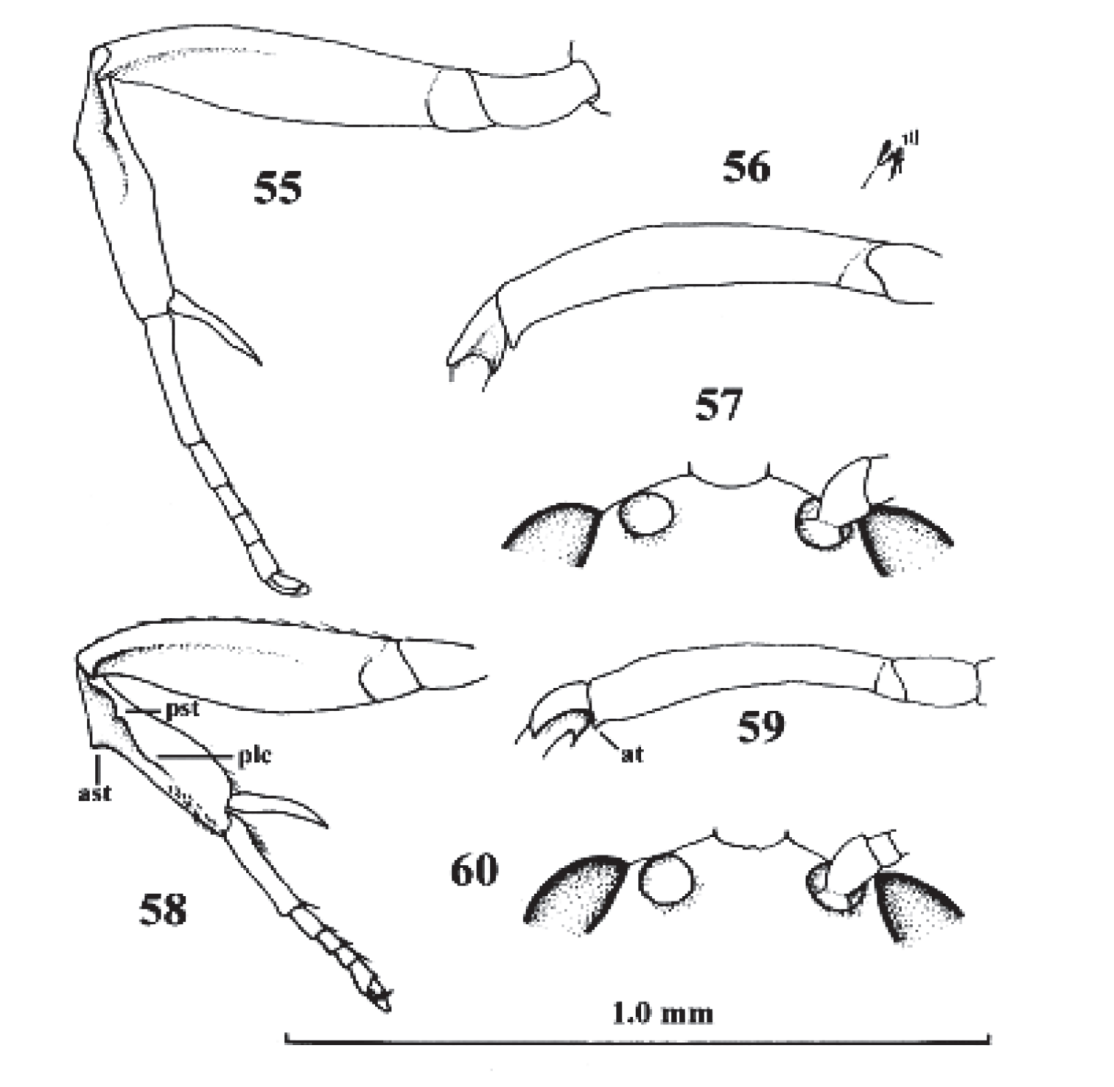
*Neoneurus recticalcar* sp. n., female, holotype. **58–60.** *Neoneurus vesculus* sp. n., female, holotype. **55, 58** fore leg inner side lateral **56, 59** fore femur dorsal **57, 60** face dorsal. 55–57 scale-line = 1.0× 58–60 1.2×. ast = anterior subbasal tooth **at** apical tooth **plc** posterior longitudinal carina **pst** posterior subbasal tooth.

Fore tibia of female about 4.8 times as long as wide, slightly narrowed basally, with short carina and below it bristly setose and with a small anterior subbasal tooth (Fig. 55); mesosoma extensively marked with pale yellowish patches (Fig. 54); metasoma brownish-yellow, with first tergite entirely blackish and most tergites basally and apically dark brown, fore femur curved in dorsal view; fore spur straight and moderately wide. It does not run in the key by Shaw (1992) to any species because of having the long spur of the fore tibia combined with short facial spines. As indicated in our key it is similar to *Neoneurus clypeatus*, but easily to separate by the nearly straight fore tibial spur and minute facial tubercles.

#### Description.

Holotype, ♀, length of body 3.3 mm, of fore wing 2.2 mm.

*Head.* Length of third segment of antenna 1.1 times fourth segment, length of third, fourth and penultimate segments 5.0,

4.7 and 7.0 times their width, respectively and basal segments without distinct setae; facial tubercles small and facial bristles 0.4 times as long as pedicellus, distance between bristles about 1.3 times width of scapus (Fig. 57); length of eye 3.2 times temple in dorsal view; vertex superficially granulate, with few superficial punctures and a satin sheen; temples directly narrowed behind eyes; OOL:diameter of ocellus:POL = 11:5:12; length of malar space 0.10 times height of eye.

*Mesosoma.* Length of mesosoma 1.4 times its height; mesoscutum superficially punctulate-granulate, but medio-posteriorly densely granulate; precoxal sulcus medially slightly impressed and with a few rugae; mesopleuron superficially granulate, but postero-dorsally shiny and largely smooth; mesosternal sulcus finely crenulate, rather narrow and moderately impressed; metanotum with a median carina, moderately protruding dorsally; propodeum finely granulate and with some rugulae, dorsal face about as long as posterior face, with satin sheen, with complete median carina and no medial areola, flat antero-medially and its spiracle small and far in front of middle of propodeum.

*Wings*. Fore wing: parastigma medium-sized (Fig. 54); basal half of wing nearly as densely setose as its distal half. Hind wing: wing membrane moderately setose basally.

*Legs.* Hind coxa largely superficially micro-granulate; fore coxa flat ventrally; all tarsal claws slender and simple; length of femur, tibia and basitarsus of hind leg 3.7, 7.6 and 7.0 times their width, respectively; fore femur curved in dorsal view, compressed and apically with medium-sized tooth; anterior subbasal tubercle of fore tibia small (Fig. 55) and longitudinal carina of tibia at basal 0.3, bearing a small posterior subbasal tooth, followed by bristly setae, area of tibia in between subbasal teeth concave (Fig. 56); fore tibia 4.7 times longer than its maximum width in lateral view; fore tibial spur straight, comparatively slender and about as long as fore basitarsus and 0.5 times fore tibia (Fig. 55); spurs of hind tibia acute apically, their length 0.8 and 0.7 times hind basitarsus.

*Metasoma.* Length of first tergite 1.6 times its apical width, its surface with satin sheen, granulate with some rugulae posteriorly, basally flat, medially convex and its spiracles slightly protruding and near middle of tergite; second tergite superficially granulate and anteriorly with some rugulae; second metasomal suture distinct but shallow; remainder of metasoma largely smooth and compressed; setae of metasoma spread, short, but second tergite and anterior half of third tergite glabrous; second tergite with sharp lateral crease; length of ovipositor sheath 0.06 times fore wing.

*Colour.* Dark brown or blackish; face (except narrow triangular patch medio-dorsally), clypeus, labrum, malar space, palpi, temple ventrally, frons anteriorly (except in front of anterior ocellus), tegulae (but humeral plate brown medially), propleuron, fore and middle coxae, trochanters and trochantelli white or ivory; four basal segments of antenna, pronotal side postero-dorsally and ventrally, remainder of legs (but hind tibia and tarsus pale brown and fore telotarsus dark brown), mesoscutum antero-laterally, mesopleuron antero-dorsally, mesosternum posteriorly, third-sixth metasomal tergites (but anteriorly and posteriorly dark brown) and seventh tergite (except a pair of oblique dark brown stripes) and eighth tergite pale yellowish; posteriorly mesoscutum with narrow W-shaped patch brown; second tergite blackish anteriorly and its posterior half brown; veins pale brown; parastigma posteriorly, pterostigma and 1-R1 largely dark brown; wing membrane slightly infuscate.

*Variation.* Length of body 3.3–3.4 mm, of fore wing 2.1–2.2 mm, all females have 16 antennal segments; distance between bristles 1.2–1.3 times width of scapus; mesoscutum sometimes with W-shaped patch medio-posteriorly and mesopleuron medially yellowish, and third (except base) and fourth antennal segments may be light brown.

#### Etymology.

From “rectus” (Latin for “straight”) and “calcar” (Latin for “spur”), because of the straight spur of the fore tibia.

### Neoneurus vesculus

van Achterberg & Gómez sp. n.

urn:lsid:zoobank.org:act:E163C037-3738-4DA9-AC35-96C884DCBF5F

http://species-id.net/wiki/Neoneurus_vesculus

Figs 58– 61

#### Type material.

Holotype, ♀ (RMNH),“Spain, Madrid, Carretera de La Coruña km 7.5, 20.viii.2010, following adult workers of *Formica cunicularia*, J.M. Gómez Durán, RMNH”. Paratypes: 11 ♀ (RMNH (8), RMSEL (2), RMS (1)), topotypic, collected 3.ix. 2010, 13.ix.2010, 24.vi.2011 and 27.vi.2011.

#### Oviposition behaviour.

In recent years the oviposition behaviour of the genus *Neoneurus* was studied for the first time (Shaw 1992, 1993) with *Neoneurus mantis* Shaw, 1992. Shaw proposed a “raptorial hypothesis” to explain the greatly modified morphology of *Neoneurus* fore legs (compression of the fore femur, robustness and shortening of the fore tibia, enlargement of the tibial spur, development of a tibial carina often associated with sharp tubercles and spines, shortening of the tarsus and enlargement of the fore pulvillus). These features, together with the two peculiar spinules situated in the frontal area of the head, could serve to grasp the ant before oviposition. Here we confirm for *Neoneurus vesculu*s sp. n. Shaw’s raptorial hypothesis. While this author mentions the metasoma of *Formica pozdzolica* as the location for alighting and oviposition for *Neoneurus mantis*, our observations show that *Neoneurus vesculus* sp. n. alights and probably oviposits in the mesosoma of *Formica cunicularia* Latreille, 1789.

The observations were made in Madrid (at the enclosed area of the Institute for Agriculture and Food Research and Technology (INIA), Carretera de La Coruña Km 7.5, Spain) during August and September, 2010, in three colonies of *Formica cunicularia* situated in the base of Atlas cedar trees (*Cedrus atlantica*). *Neoneurus vesculus* sp. n. could be seen around the nest entrances in the morning and in the afternoon, with a peak activity of oviposition attacks between 4–7 PM. Two strategies were observed (Fig. 62): a) the perching behaviour as described by Shaw (1993), the wasp standing on a grass stem, on a tree trunk (in both cases at a height less than 5 cm), or on the ground, until an ant approaches; a moment later the wasp takes flight and begins its attack (Movie *Neoneurus*, first sequence, Appendix III); b) the hovering behaviour -at a distance of about 1 cm- over ants leaving the nest entrance and going up the tree trunk at a height of 3 or 4 cm from the ground. Oviposition attacks following hovering behaviour (Movie *Neoneurus*, second sequence) increased in the afternoon, being then predominant over the perching behaviour.

When the ant moves up, the wasp approaches it from behind and waits until the ant’s body is in a vertical position. Then, the wasp head hits the ant’s mesonotum while the fore legs dart forward and brace the mesopleuron. The frame analysis reveals that the tibia are the part of the legs that firmly hold the mesopleuron (Figs 63-65).

After contact, the wasp’s head is separated from the ant’s body, the wasp’s metasoma is placed vertically and its wings are folded. Then ovipositor insertion begins, during which time the middle legs can be seen to be sometimes holding the posterior part of the ant’s mesosoma (Fig. 64). The wasp’s metasoma is bent towards the posterior lower part of the ant’s mesosoma, going between the metasoma and the hind leg of the ant. This occurred in a surprisingly asymmetric fashion: of 29 ovipositions observed, the wasp always bent its metasoma between the left hind leg of the ant and the left side of its metasoma. This suggests some asymmetrical morphology of the ovipositor system. According to the frame analysis, the ovipositor was inserted near the posterior coxal cavities, perhaps into the coxal cavities of the middle or hind legs or in the area between them (Fig. 66).

Oviposition was not always fully successful. Of a total of 25 attempts observed, 17 were completed, 4 were initiated but ended with the wasp and the ant -still joined- falling to the ground, and in the other 4 cases the wasp failed to grasp the ant and flew away immediately. Hence, the grasping of the ant appears to be a critical moment of the oviposition process. Sometimes the wasp’s head hit on the ant’s pronotum instead of its mesonotum, or the wasp attacked an ant that was not in a vertical position. In these circumstances it had more difficulty holding the ant, whose vigorous movements usually resulted in oviposition failure. Other times the first hit of the wasp’s head, together with the strong grasping of its fore legs, caused the ant to detach from the surface and fall down with the wasp.

The whole oviposition behaviour of *Neoneurus vesculus* sp. n. (comprising the grasping of the ant by the wasp and the insertion of the ovipositor, until taking flight) lasted a mean of 2.023 seconds (95% confidence interval: 1.352–2.694; N = 17; SE = 0.317), with a median of 1.507 seconds (interquartile range: 1.377–1.927; Fig. 79). Three outlier observations corresponded to ovipositions lasting more than 3 seconds due to the ant’s vigorous movements which made it difficult for the wasps to bend their metasomata towards the postero-lower part of the ants’ mesosomata.

On one occasion a strange behaviour was observed. One *Neoneurus* hovering over the nest entrance alighted on the tree trunk, turning and resting, 2 cm away from a worker. The ant approached and touched the apex of the wasp’s metasoma with its antenna. Then the wasp curved its metasoma inward extruding the ovipositor. Finally, the ant attacked the wasp, held it by the wings, and transported it into the nest.

#### Discussion.

The described oviposition behaviour of *Neoneurus vesculus* sp. n. fits well with the raptorial function predicted by Shaw for the modified morphology of the genus *Neoneurus*. The head spinules may fix the position of the wasp when its head hits the ant’s mesonotum and the robust tibia are suitable for grasping the ant’s mesosoma by the mesopleura. The location of the wasp when alighting on the ant, and the final arrangement of its body, allow the insertion of the ovipositor into the postero-lower part of the ant’s mesosoma. These facts call for a re-examination, with high speed photography or video, of the oviposition behaviour of *Neoneurus mantis* in order to confirm the alighting and oviposition of this species in the ant metasoma, as mentioned by Shaw (1993). It may be remarked that this author several times dissected the ant’s metasoma following the wasp’s oviposition, and could not find the wasp’s eggs. The possibility is open that *Neoneurus mantis*, and other species of the genus, have a similar oviposition behaviour to that of *Neoneurus vesculus* sp. n., and hence that the eggs are laid in the ant’s mesosoma.

#### Diagnosis.

Fore tibia of female about 4.0 times as long as wide, distinctly narrowed basally, with long carina and below it a double row of small pegs and with a wide triangular anterior subbasal tooth; mesosoma extensively marked with pale yellowish patches; metasoma brownish-yellow, with first tergite entirely blackish and most tergites basally and apically dark brown, fore femur curved in dorsal view; fore spur nearly straight and robust; facial tubercles small and facial bristles 0.2 times as long as pedicellus, distance between bristles about 1.2 times width of scapus. Runs in the key by Shaw (1992) to *Neoneurus pallidus* Shaw, 1992, from Canada (Ontario) and USA (Maryland, Michigan, North Carolina, Virginia and Colorado), but the new species has the third and fourth antennal segments pale yellowish (dark brown (except base of third segment) in *Neoneurus pallidus*); first metasomal tergite entirely blackish (dark yellowish-brown and medially irregularly black), apex and base of second-sixth tergites dark brown (entirely pale yellowish-brown except dark yellowish-brown base of second tergite), fore tibia with row of small pegs below carina (below carina largely smooth, but a few small pegs near apex), fore tibia of female 4.0 times longer than its maximum width in lateral view (4.5 times) and fore tibia of female distinctly narrowed basally (slightly narrowed; Fig. 4 in Shaw 1992).

#### Description.

Holotype, ♀, length of body 2.8 mm, of fore wing 1.8 mm.

*Head.* Length of third segment of antenna 1.1 times fourth segment, length of third, fourth and penultimate segments 5.3, 4.8 and 2.5 times their width, respectively and basal segments without distinct setae; facial tubercles small and facial bristles 0.2 times as long as pedicellus, distance between bristles about 1.2 times width of scapus (Fig. 60); length of eye 1.5 times temple in dorsal view; vertex superficially granulate, with few superficial punctures and a satin sheen; temples directly narrowed behind eyes; OOL:diameter of ocellus:POL = 6:3:7; length of malar space 0.13 times height of eye.

*Mesosoma.* Length of mesosoma 1.4 times its height; mesoscutum superficially punctulate-granulate, but medio-posteriorly densely granulate; precoxal sulcus only medially impressed and with a few rugae; mesopleuron superficially granulate, but postero-dorsally shiny and largely smooth; mesosternal sulcus finely crenulate, narrow and rather shallow; metanotum with a median carina, not protruding dorsally; propodeum finely granulate and with some rugulae, dorsal face longer than posterior face, with satin sheen, only dorsally with a median carina and no medial areola, flat antero-medially and its spiracle small and far in front of middle of propodeum.

**Figure 61.**
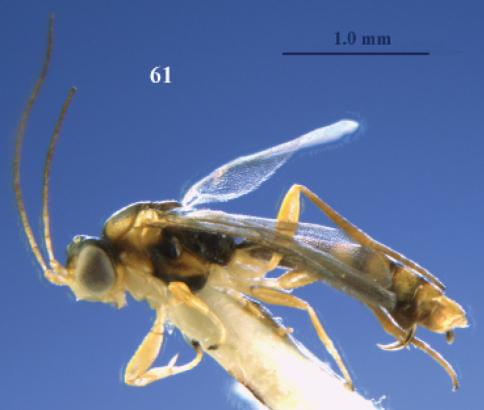
*Neoneurus vesculus* sp. n., female, holotype. Habitus lateral.

*Wings.* Fore wing: parastigma medium-sized (Fig. 61); basal half of wing nearly as densely setose as its distal half. Hind wing: wing membrane moderately setose basally.

*Legs.* Hind coxa nearly smooth, dorsally partly superficially micro-granulate; fore coxa flat ventrally; all tarsal claws slender and simple; length of femur, tibia and basitarsus of hind leg 3.9, 9.2 and 5.3 times their width, respectively; fore femur curved in dorsal view, compressed and apically with small tooth; anterior subbasal tubercle of fore tibia wide triangular (Fig. 58) and longitudinal carina of tibia at basal 0.6, bearing a small posterior subbasal tooth and apical half curved, followed by a row of small slender pegs, area of tibia in between subbasal teeth concave (Fig. 59); fore tibia 4.0 times longer than its maximum width in lateral view; fore tibial spur nearly straight and 0.9 times as long as fore basitarsus and 0.5 times fore tibia (Fig. 58); spurs of hind tibia acute apically, their length 0.7 and 0.6 times hind basitarsus.

*Metasoma.* Length of first tergite 1.4 times its apical width, its surface with satin sheen, granulate with some rugulae posteriorly, basally flat, medially convex and its spiracles slightly protruding and near middle of tergite; second tergite superficially granulate and anteriorly with some oblique rugulae; second metasomal suture obsolescent; remainder of metasoma largely smooth and compressed; setae of metasoma spread, short, but tergites glabrous anteriorly; second tergite with sharp lateral crease; length of ovipositor sheath 0.05 times fore wing.

*Colour.* Dark brown or blackish; face, clypeus, labrum, malar space, temple ventrally, pronotal side postero-dorsally and ventrally, frons antero-laterally, propleuron, palpi, coxae, trochanters and trochantelli white or ivory; four basal segments of antenna, remainder of legs (but hind tibia and tarsus brown and telotarsi dark brown), tegulae, mesoscutum antero-laterally and a W-shaped patch posteriorly, scutellum (except dark medial patch), mesopleuron antero-dorsally and medially, mesosternum posteriorly, second-fifth metasomal tergites (but anteriorly and posteriorly dark brown) and sixth-eighth tergites pale yellowish; veins pale brown; parastigma, pterostigma and 1-R1 largely dark brown; wing membrane slightly infuscate.

*Variation.* Length of body 2.6–3.0 mm, of fore wing 1.8–1.9 mm, all females have 16 antennal segments; mesoscutum medially, mesopleuron antero-medially and scutellum may be dark brown; third and fourth antennal segments pale yellow or brownish.

#### Etymology.

 From “vesculus” (Latin for “weak, little, poor”) because this new species has poorly developed facial bristles.

**Figure 62.**
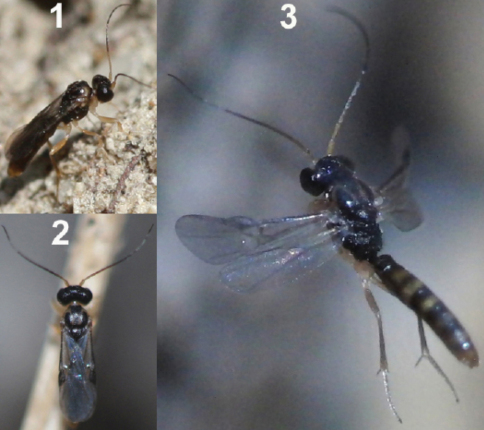
Female of *Neoneurus vesculus*sp. n. **1** standing on the ground **2** on a grass stem and **3** hovering over the nest entrance.

**Figure 63.**
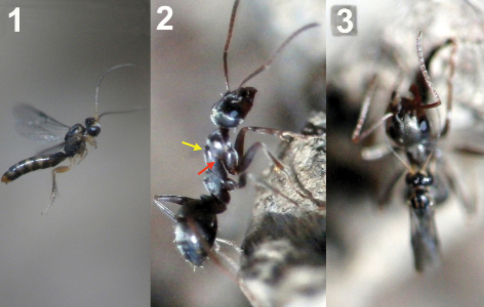
**1** female of *Neoneurus vesculus*sp. n. hovering over the nest entrance **2** worker of *Formica cunicularia* showing the mesonotum (yellow arrow) where the wasp’s head will hit, and the mesopleuron (red arrow) that will be braced by the wasp’s legs **3** after the first hit, the wasp’s metasoma is positioned vertically and its wings are folded.

**Figure 64.**
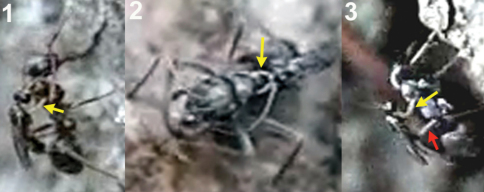
Position of wasp’s tibiae (yellow arrow) of three *Neoneurus vesculus* sp. n. while bracing the ant’s mesopleuron. In frame **3** the middle legs can be appreciated (red arrow) grasping the posterior part of the mesosoma just before the insertion of the ovipositor.

**Figure 65.**
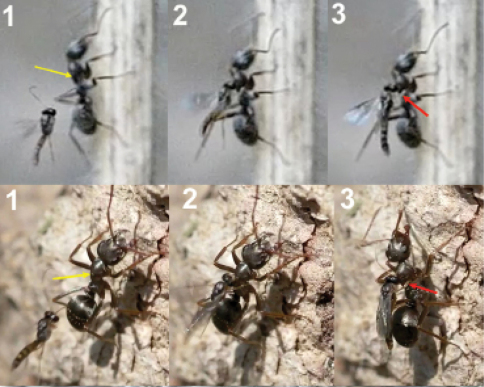
Two attack sequences of *Neoneurus vesculus*sp. n. **1** approaching a worker of *Formica cunicularia* and fixing its attention on the ant’s mesonotum (yellow arrow) **2** hitting its head on the ant’s mesonotum and extending the fore legs **3** bracing the ant’s mesopleuron with its fore tibia (red arrow), then placing the metasoma vertically, parallel to the ant’s body, and folding the wings prior to oviposition.

**Figure 66.**
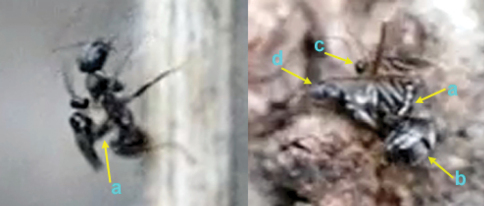
Insertion of the ovipositor by *Neoneurus vesculus*sp. n. **a** wasp metasoma **b** ant metasoma **c** wasp head; **d** ant head.

**Figures 67–71.**
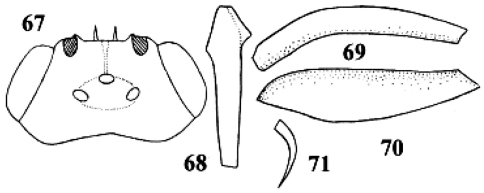
*Neoneurus curvicalcar* Belokobylskij, female, holotype. **67** head dorsal **68** fore tibia inner side lateral **69** fore femur dorsal **70** fore femur lateral **71** fore tibial spur. After Belokobylskij (1986).

## Ichneumonidae Latreille, 1802

### Hybrizon

Fallén, 1813

http://species-id.net/wiki/Hybrizon

Hybrizon Fallén, 1813: 19 (no species). Type species (by subsequent monotypy, first included species): *Hybrizon latebricola* Nees, 1834 (= *Hybrizon buccatus* (de Brébisson, 1825)).

#### Notes.

For a key to the European species, see van Achterberg (1999).

### Hybrizon buccatus

(de Brébisson, 1825)

http://species-id.net/wiki/Hybrizon_buccatus

**Figure 72. F33:**
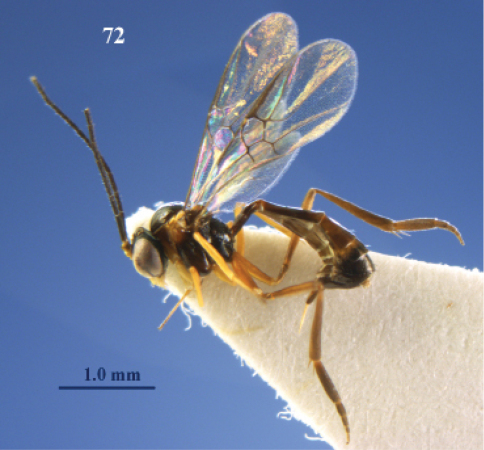
*Hybrizon buccatus* (de Brébisson), female, Spain, Madrid. Habitus lateral.

Figs 72 [Fig F33] [Fig F34] [Fig F35] [Fig F36] [Fig F37] 78

Paxylomma buccata de Brébisson, 1825: 23 (type lost).

#### Oviposition behaviour.

Donisthorpe and Wilkinson (1930) found naked pupae of *Hybrizon buccatus* among the cocoons of the ant *Lasius alienus*, and concluded that *Hybrizon buccatus* is likely to be parasitoid of adult ants, as are the wasps of the genus *Elasmosoma*. Although authors such as Watanabe (1984) and Marsh (1989) suggested that *Hybrizon* species may be endoparasitoids of ant larvae, the adult-parasitism hypothesis has remained, being included in general revisions dealing with ants (Hölldobler and Wilson 1990; Schmid-Hempel 1998). Here we report larval-parasitism of *Hybrizon buccatus* of the ant *Lasius grandis* Forel, 1909.

**Figure 73. F34:**
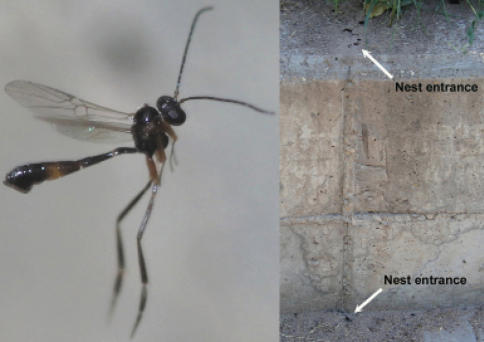
Female of *Hybrizon buccatus* (left) hovering over a permanent vertical trail established between two nest entrances of a colony of *Lasius grandis* (right).

The observations were made in Almazán (Soria, Spain) during July and August, 2010, on a permanent vertical trail of *Lasius grandis*, situated on a wall 60 cm high. The ants walked up and down, day and night, between two nest entrances of the same colony, one placed in the base and the other at the top of the wall (Fig. 73).

**Figure 74. F35:**
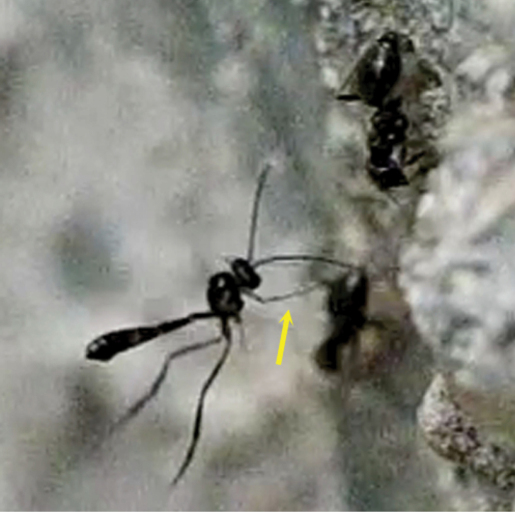
Female of *Hybrizon buccatus* approaches an ant and touches the metasoma with its fore leg (yellow arrow). Immediately, the wasp retreats the leg. No oviposition takes place.

During the 3 weeks of observations, especially between 5–8 PM, one or two females of *Hybrizon buccatus* could be seen hovering over the trail, at 1 cm or less from the ants, usually in the lower part of the trail (less than 15 cm from the base). They could remain almost stationary in the air for more than 5 minutes. Even in the absence of ants on the trail for a period of time, specimens of *Hybrizon buccatus* found the precise location of the trail and stayed hovering over it. Location of the trail may involve olfactory or visual clues or both. After a long set of video recording, and hundreds of workers passing through the trail, no oviposition of the wasp could be observed on adult ants. Sometimes the wasp followed and approached an ant with a very quick movement, even touching the ant metasoma with its fore legs, but without oviposition (Fig. 74). This rapid approaching behaviour must be the one referred to Giraud (1857) and Donisthorpe (1910), respectively, as “pounces” and “striking” at the ants, behaviour that led Donisthorpe and Wilkinson 1930) to conclude adult-parasitism for *Hybrizon buccatus*.

**Figure 75. F36:**
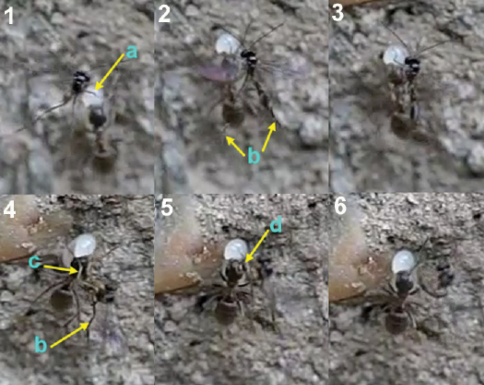
Oviposition sequence of *Hybrizon buccatus*. **a** fore legs **b** hind legs **c** middle legs **d** apex of the metasoma. In frame **4** the wasp begins to bend the metasoma and folds the wings. In frame **5** the apex of the metasoma reaches the ant larva and oviposition takes place.

The analyse of video frames revealed oviposition of *Hybrizon buccatus* into the final instar larvae of *Lasius grandis* while being transported by worker ants. Two cases were recorded, one with the worker going upward, and the other with the worker going downward (Movie *Hybrizon*, Appendix IV). In the first case (Fig. 75) the wasp grasped the larva with its fore legs and placed its body in a vertical position over the adult ant. When the metasoma began to bend toward the larva, the middle legs seized the adult ant’s head, and the wings were folded until oviposition finished. Throughout the process the hind legs remained in the air. The whole behaviour, comprising the grasping of the larva and the insertion of the ovipositor, until flying off, lasted 0.40 seconds.

**Figure 76. F37:**
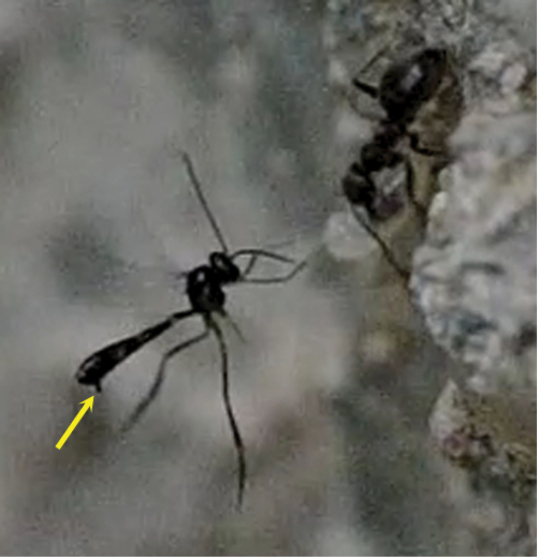
The arrow points the exserted ovipositor of the female of *Hybrizon buccatus* while the fore legs grasp the ant larva.

In the second case (Fig. 76), contact of the fore legs with the larva can be seen, while the ovipositor is exserted. The middle legs are probably used to grasp the larva during the bending of the metasoma and oviposition. Again, the hind legs hang in the air. The whole behaviour lasted 0.58 seconds.

**Figure 77.**
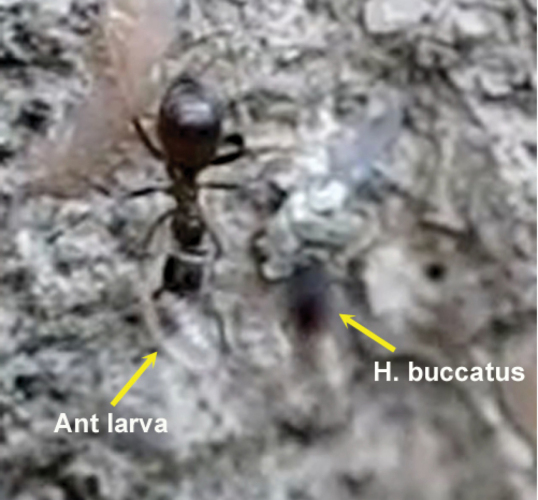
Female of *Hybrizon buccatus* ignores a smaller ant larva transported by a worker.

Specimens of *Hybrizon buccatus* twice ignored smaller larvae transported by workers of *Lasius grandis* (Fig. 77), which may indicate that only final instar larvae are selected for oviposition.

**Figure 78.**
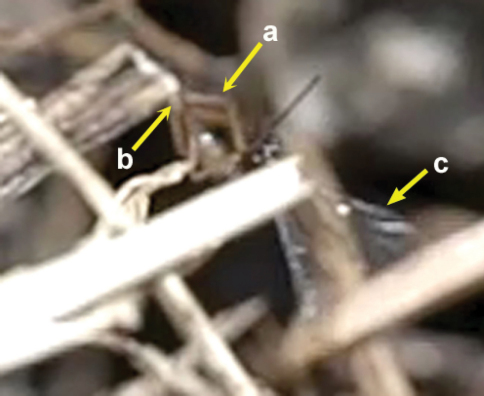
Aberrant behaviour of a female of *Hybrizon buccatus*. **a** legs **b** apex of the metasoma touching the grass stem **c** wings.

An unexplained aberrant behaviour was observed in Madrid (at the enclosed area of the Institute for Agriculture and Food Research and Technology (INIA), Carretera de La Coruña Km 7.5, Spain) in September, 2010, when a female of *Hybrizon buccatus* was hovering near a nest of *Lasius grandis* located at the base of an Atlas cedar tree (*Cedrus atlantica*). First, the wasp held the apex of a grass stem with its fore legs, and then grabbed it with all legs, bending the metasoma and folding the wings (last sequence of Movie *Hybrizon* and Fig. 78). The frame analysis revealed the movement of the apex of the wasp metasoma touching the stem. The whole behaviour lasted 0.30 seconds.

**Figure 79.**
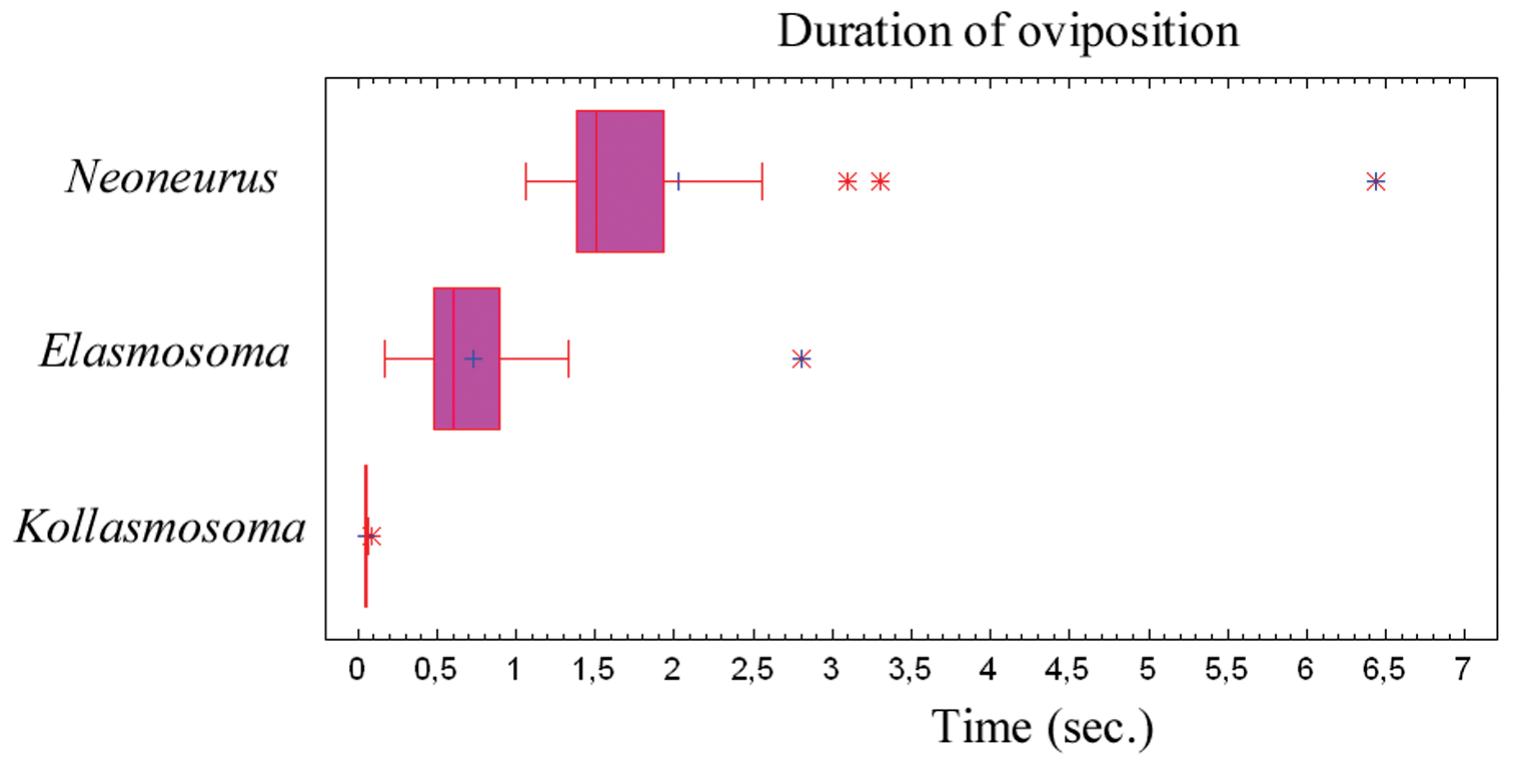
Duration of the oviposition behaviour (comprising the grasping of the ant by the wasp and the insertion of the ovipositor, until taking off) of three neoneurine Euphorinae: *Neoneurus vesculus* sp. n. (N = 17), *Elasmosoma luxemburgense* (N = 38) and *Kollasmosoma sentum* sp. n. (N = 19). Interquartile ranges and outlier data are given.

## General conclusions

From the observations here recorded on the oviposition behaviour of four European ant parasitoid wasps, some general conclusions are offered. The grasping of the ant (or the larva, in the case of *Hybrizon buccatus*) appears to be a critical phase of the wasps’ oviposition. In all four species the wings are folded after alighting on the ant and during the insertion of the ovipositor. The legs are used to grasp the ant’s body, following different strategies according to the species. *Hybrizon buccatus* uses the fore and middle legs to hold the ant larva; *Neoneurus vesculus*sp. n. has especially adapted fore legs to grasp the ant mesosoma firmly, making secondary use of the middle and, probably, hind legs. *Elasmosoma luxemburgense* and *Kollasmosoma sentum* sp. n. use all three pairs of legs. In all four species the fore legs are the first to grasp the ant (or the larva, in the case of *Hybrizon buccatus*). *Neoneurus vesculus*sp. n., and usually *Elasmosoma luxemburgense*, hit the ant’s body with their heads when alighting.

In order to grasp the host, the visual perception of these ant parasitoids seems highly developed, especially considering the extremely short time elapsing during the oviposition sequence (Fig. 79). *Hybrizon buccatus* detects the ant larvae transported by workers along the trails, most probably selecting the mature instars. *Neoneurus vesculus* sp. n.directly hits with its head on the ant’s mesonotum. *Elasmosoma luxemburgense* tends to alight by fixing attention on the posterior margin of the first gastral segment of the ant. *Kollasmosoma sentum* sp. n. has different alighting strategies corresponding to the inclination of the host’s (*Cataglyphis*) metasoma, always aligning itself with the longitudinal axis of the ant’s metasoma.

The location of the oviposition insertion varies in the four species, each presenting particular situations. *Hybrizon buccatus* lays the egg into an ant larva, apparently without any locational preference, but with the difficulty of dealing with the moving transporter worker. *Neoneurus vesculus* lays the egg in the postero-ventral part of the ant’s mesosoma, bending its metasoma between the hind leg of the ant and the metasoma, certainly the longer and most complex of the ovipositions observed. *Elasmosoma luxemburgense* lays an egg into the posterior area of the last metasomal segment, probably through the anus. *Kollasmosoma sentum* lays the egg in any location of the ant’s metasomal surface, probably through an intersegmental membrane; its extremely fast oviposition seems well adapted to the very speedy workers of *Cataglyphis ibericus*, which usually march with the metasoma held in a vertical position.

Regarding the oviposition behaviour of the three neoneurines, the persistent defensive behaviour displayed by the ants is also significant. The ants are usually aware of the presence of the wasps, to which they turn towards with opened mandibles and sometimes catch them. Oviposition is also frequently impeded by the hits and movements of the ant’s legs.

## Supplementary Material

XML Treatment for Elasmosoma luxemburgense

XML Treatment for Kollasmosoma

XML Treatment for Kollasmosoma sentum

XML Treatment for Neoneurus

XML Treatment for Neoneurus auctus

XML Treatment for Neoneurus clypeatus

XML Treatment for Neoneurus recticalcar

XML Treatment for Neoneurus vesculus

XML Treatment for Hybrizon

XML Treatment for Hybrizon buccatus

## Appendix I

Movie *Elasmosoma*. (doi: 10.3897/zookeys.125.1754.app1) File format: MOV

**Explanation note:** Females of the parasitoid wasp *Elasmosoma luxemburgense* ovipositing in workers of the ant *Formica rufibarbis*. In the last sequence a worker ant catches a wasp while flying. Recorded in slow motion video, at a rate of 300 frames per second. Almazán (Soria, Spain), August, 2010.

Copyright notice: This dataset is made available under the Open Database License (http://opendatacommons.org/licenses/odbl/1.0/). The Open Database License (ODbL) is a license agreement intended to allow users to freely share, modify, and use this Dataset while maintaining this same freedom for others, provided that the original source and author(s) are credited.

## Appendix II

Movie *Kollasmosoma*. (doi: 10.3897/zookeys.125.1754.app2) File format: MOV

**Explanation note:** Females of the parasitoid wasp *Kollasmosoma sentum* ovipositing in workers of the ant *Cataglyphis ibericus*. Recorded in slow motion video, at a rate of 300 frames per second. Madrid, August and September, 2010.

Copyright notice: This dataset is made available under the Open Database License (http://opendatacommons.org/licenses/odbl/1.0/). The Open Database License (ODbL) is a license agreement intended to allow users to freely share, modify, and use this Dataset while maintaining this same freedom for others, provided that the original source and author(s) are credited.

## Appendix III

Movie *Neoneurus*. (doi: 10.3897/zookeys.125.1754.app3) File format: MOV

**Explanation note:** Females of the parasitoid wasp *Neoneurus vesculus* ovipositing in workers of the ant *Formica cunicularia*. Recorded in slow motion video, at a rate of 300 frames per second. Madrid, August and September, 2010.

Copyright notice: This dataset is made available under the Open Database License (http://opendatacommons.org/licenses/odbl/1.0/). The Open Database License (ODbL) is a license agreement intended to allow users to freely share, modify, and use this Dataset while maintaining this same freedom for others, provided that the original source and author(s) are credited.

## Appendix IV

Movie *Hybrizon*. (doi: 10.3897/zookeys.125.1754.app4) File format: MOV

**Explanation note:** Females of the parasitoid wasp *Hybrizon buccatus* ovipositing in the ant larvae transported by workers of *Lasius grandis*. In the last sequence a female of *Hybrizon buccatus* holds the tip of a grass stem with its legs, then bends the metasoma and touches the grass stem with its metasomal apex. Recorded in slow motion video, at a rate of 300 frames per second. Almazán (Soria, Spain), July and August, 2010.

Copyright notice: This dataset is made available under the Open Database License (http://opendatacommons.org/licenses/odbl/1.0/). The Open Database License (ODbL) is a license agreement intended to allow users to freely share, modify, and use this Dataset while maintaining this same freedom for others, provided that the original source and author(s) are credited.
